# Intestinal cancer stem cells marked by Bmi1 or Lgr5 expression contribute to tumor propagation via clonal expansion

**DOI:** 10.1038/srep41838

**Published:** 2017-02-08

**Authors:** Hirotsugu Yanai, Naho Atsumi, Toshihiro Tanaka, Naohiro Nakamura, Yoshihiro Komai, Taichi Omachi, Kiyomichi Tanaka, Kazuhiko Ishigaki, Kazuho Saiga, Haruyuki Ohsugi, Yoko Tokuyama, Yuki Imahashi, Shuichi Ohe, Hiroko Hisha, Naoko Yoshida, Keiki Kumano, Masanori Kon, Hiroo Ueno

**Affiliations:** 1 Department of Stem Cell Pathology, Kansai Medical University, 2-5-1 Shin-machi, Hirakata, Osaka 573-1010, Japan; 2 Department of Surgery, Kansai Medical University, 2-5-1 Shin-machi, Hirakata, Osaka 573-1010, Japan; 3 Department of Internal Medicine, Kansai Medical University, 2-5-1 Shin-machi, Hirakata, Osaka 573-1010, Japan; 4 Department of Urology and Andrology, Kansai Medical University, 2-5-1 Shin-machi, Hirakata, Osaka 573-1010, Japan; 5 Department of Pediatrics, Kansai Medical University, 2-5-1 Shin-machi, Hirakata, Osaka 573-1010, Japan; 6 Department of Dermatology, Kansai Medical University, 2-5-1 Shin-machi, Hirakata, Osaka 573-1010, Japan

## Abstract

Although the existence of cancer stem cells in intestine tumors has been suggested, direct evidence has not been yet provided. Here, we showed, using the multicolor lineage-tracing method and mouse models of intestinal adenocarcinoma and adenoma that Bmi1- or Lgr5- positive tumorigenic cells clonally expanded in proliferating tumors. At tumor initiation and during tumor propagation in the colon, the descendants of Lgr5-positive cells clonally proliferated to form clusters. Clonal analysis using ubiquitous multicolor lineage tracing revealed that colon tumors derived from Lgr5-positive cells were monoclonal in origin but eventually merged with neighboring tumors, producing polyclonal tumors at the later stage. In contrast, the origin of small intestine tumors was likely polyclonal, and during cancer progression some clones were eliminated, resulting in the formation of monoclonal tumors, which could merge similar to colon tumors. These results suggest that in proliferating intestinal neoplasms, Bmi1- or Lgr5-positive cells represent a population of cancer stem cells, whereas Lgr5-positive cells also function as cells-of-origin for intestinal tumors.

The cancer stem cell theory has gained considerable attention among oncologists, as it describes a cell population responsible for cancer initiation and progression, thus revealing a prospective target for anti-cancer treatment. Polycomb complex protein (Bmi1) and leucine-rich-repeat containing G-protein-coupled receptor 5 (Lgr5) have been identified as molecular markers of multipotent adult stem cells in the small intestine, which promote regeneration of the intestinal epithelium and represent the cells-of-origin in intestinal cancer^1^,^2^,^3^. However, it is unclear whether the expression of these proteins persists in cancer stem cells of proliferating tumors and whether it can be used for the detection of stem cell populations in progressing intestinal cancer. Here, we employed multicolor lineage tracing^4^,^5^,^6^ to reveal the contribution of Bmi1- or Lgr5-positive tumorigenic cells to the propagation of intestinal tumors. The model was based on an inducible system using Cre recombinase fused to a mutated form of the ligand-binding domain of the estrogen receptor (ERT2) with affinity to tamoxifen. This system can label cells that express the gene of interest by randomly inducing the expression of one of four different fluorescent proteins, and the color pattern of the formed tumors would indicate their ability to clonal expansion.

A multistep hit model, which faithfully reproduces pathogenesis of human colon carcinoma, has been proposed to explain the development of colon cancer, where benign adenoma is first formed and then the mutation of specific genes drives carcinogenesis^7^. To mimic the progression of adenoma to carcinoma, we used a two-step carcinogenesis model based on mice carrying the mutation in the gene encoding adenomatous polyposis coli (*Apc*^*min*^) and treated with dextran sodium sulfate (DSS)^8^. *Apc*^*min*/+^ mice develop multiple intestinal tumors, mostly in the small intestine at a young age and are used as the model for familial adenomatous polyposis (FAP)^9^. In addition, we created a sporadic carcinogenesis mouse model by treating mice with azoxymethane followed by DSS^10^. The use of FAP and two intestinal cancer models enabled us to investigate the commitment of cancer stem cells in intestinal tumors developed by different mechanisms. The results of this study show that all types of intestinal tumors contain Bmi1- or Lgr5-positive cells, which clonally expand to contribute to tumor propagation.

## Results

### Bmi1+ cells in proliferating intestine tumors clonally expand

**Figure 1:**
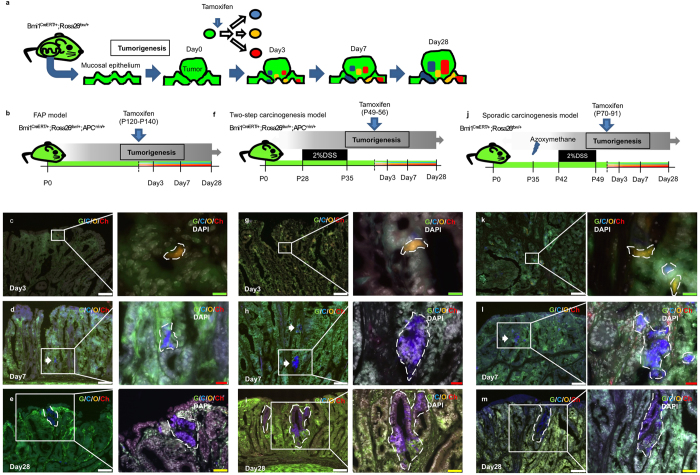
Lineage-tracing using tamoxifen-inducible multicolor labeling system to track the cell-fate of Bmi1-positive tumorigenic cells in various models of intestinal tumors. (**a**) Schematic protocol of the CreERT2-mediatid multicolor labeling using *Bmi1*^*CreERT*/+^; *Rosa26*^*rbw*/+^ mice. Rainbow (rbw) construct is knocked-in into Rosa26 locus and consists of three loxP variants: lox2271, loxN, and loxP, and subsequent cDNA for Green Fluorescent protein (GFP) followed by cDNAs for mCerulean, mOrange and mCherry, which are proceeded by lox2271, loxN, and loxP, respectively. This mouse was crossed with *Bmi1*^*CreERT*/+^ line for the purpose of multicolor lineage tracing of Bmi1-positive cells. When this mouse, all the cells of which express GFP, received tamoxifen, recombination is occurred in Bmi1-positive cells mediated by CreERT2, leading to GFP deletion and random exercise between two lox2271s, loxNs, or loxPs. As a result, mCerulean, mOrange or mCherry moves next to CAG promoter, resulting in random expression of either fluorescent protein. After tumor was confirmed to occur, the mice were injected with tamoxifen to induce multicolor labelling. (**b**) Schematic protocol of lineage-tracing of Bmi1-positive cells in tumor-bearing FAP model mice. FAP spontaneously occurs in *APC*^*min*/+^ mice before the age of 140. P, Postnatal day. (**f**) Schematic protocol of lineage-tracing of Bmi1-positive cells in tumor-bearing two-step carcinogenesis model mice. When *APC*^*min*/+^ mice, which develop FAP, are administrated with 2% DSS from the age of 28 to 35, tumorigenesis is accelerated to generate colon adenoma. P, Postnatal day. (**j**) Schematic protocol of lineage-tracing of Bmi1-positive cells in tumor-bearing sporadic carcinogenesis model mice. P, Postnatal day. (**c–e,g–i,k–m**) Representative traced images of Bmi1+ cell induced in developing tumors. Tumors were harvested at 3 days after induction (**c,g** and **k**), 7 days after induction (**d,h and l**) and 28 days after induction (**e,i and m**) in FAP model, two-step carcinogenesis model, and sporadic carcinogenesis model, respectively. G; GFP, C; mCerulean, O; mOrange, Ch; mCherry. Scale bars, white; 100 μm, yellow; 50 μm, red; 20 μm, green; 10 μm.

**Figure 2:**
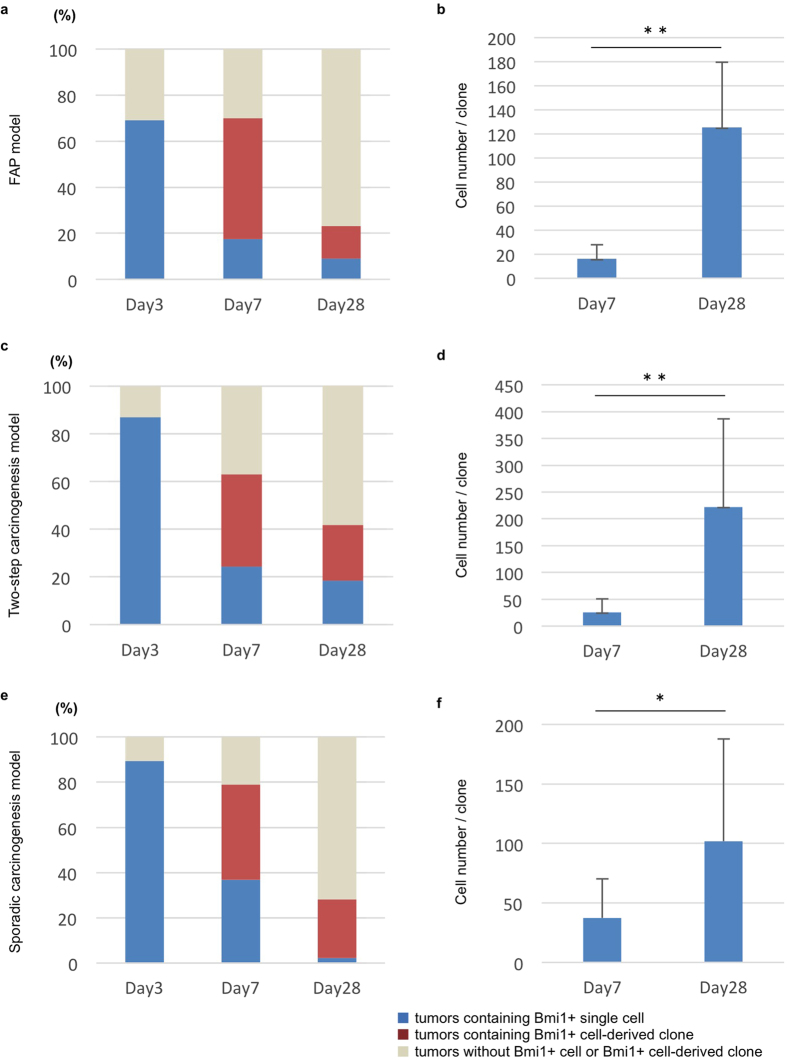
Existence and manner of clonal expansion of Bmi1+ cells in developing small intestinal tumors and colon tumors. (**a,c,e**) To assess the existence of Bmi1+ cells in developing tumor and the percentage of the tumors containing Bmi1+ cell-derived clone, FAP model (**a**), two-step caricinogenesis model (**c**), and sporadic carcinogenesis model (**e**) were set up using *Bmi1*^*CreERT*/+^*; Rosa26*^*rbw*/+^ mice. The percentage of the developing tumors that contained Bmi1+ positive cells was calculated by tamoxifen induction of tumor-bearing mice followed by 3-day chase and observation of the cells with rainbow color; mCerulean, mOrange, and mCherry (Day3). To evaluate the percentage of the tumors containing Bmi1+ cell-derived clone, tumor-bearing mice of three models were injected with tamoxifen and clones with rainbow color were examined at 7 days or 28 days after induction (**a,c,e)**: Day7, and Day28. At the same time, tumors that contained rainbow-colored single cell, which was suggested to express Bmi1 when tamoxifen induction but had not divided, was categorized as “tumors containing Bmi1+ single cell”. The number of mice and tumors analyzed were shown in Supplementary Table 1. (**b,d,f**) The number of the cells that comprised each Bmi1+ cell-derived clone were measured, and the average is shown. Cell number per clone at day28 after tamoxifen induction was compared with day7. Error bars indicate standard deviation. **p < 0.01, *p < 0.05. The number of tumors analyzed and the raw data are shown in Supplementary Table 2.

To examine the presence of Bmi1+ tumorigenic cells and their contribution to tumor progression in three models, we labeled Bmi1-positive cells with four different fluorescent proteins and performed lineage tracing (Fig. 1a). FAP and two-step carcinogenesis models were generated using *APC*^*min*/+^, *Bmi1*^*CreERT*/+^, and *Rosa26*^*rbw*/+^ mouse strains (Fig. 1b, and f)^4^,^5^,^6^. When Cre-mediated recombination occurred after tamoxifen induction, Bmi1-positive cells expressed mCerulean, mCherry, or mOrange instead of original GFP (Fig. 1a)^4^. In this multicolor lineage-tracing system, the expression of a fluorescent protein was passed from parental cells to their descendants; therefore, cells expressing the same fluorescent protein would belong to a single Bmi1-positive cell-derived clone. By inducing mice carrying developed tumors with tamoxifen, we labeled Bmi1-positive tumorigenic cells. To generate a sporadic carcinogenesis model, *Bmi1*^*CreERT*/+^; *Rosa26*^*rbw*/+^ mice were treated with azoxymethane and DSS and then were injected tamoxifen (Fig. 1j). For all models, tumor areas were defined as those containing the cells whose β-catenin expression was localized to their nuclei (Supplementary Fig. 1). At day 3 after induction, most tumors contained Bmi1+ cells (although their number in each model was small) (Fig. 1c,g and k). Thus, 69%, 87%, and 89.4% tumors in the FAP, two-step carcinogenesis, and sporadic carcinogenesis, respectively, models had Bmi1+ cells (Fig. 2a,c,e and Supplementary Table 1). At day 7 after induction, mice in all three models developed tumors containing Bmi1+ cell-derived clones (Fig. 1d,h and 1): 52.5%, 38.7%, and 42.1%, respectively (Fig. 2a,c,e and Supplementary Table 1). At day 28, the percentage decreased in each model (Fig. 2a,c,e, and Supplementary Table 1), but the size of Bmi1+ cell-derived clones significantly increased compared to that at day 7 (Figs 1e,i,m and 2b,d,f and Supplementary Table 2). On the other hand, single fluorescent cells that expressed Bmi1 at the time of tamoxifen injection but did not divide thereafter were observed both at days 7 and 28 after induction in all three models (Fig. 2a,c,e and Supplementary Table 1), indicating that not all Bmi1+ cells in tumors behaved as cancer stem cells. It should be noted that none of the tumors exhibited mixed populations of cells labeled with different colors at day 28; instead, neighboring clones could be clearly distinguished, consisting of definite numbers of cells (Fig. 1e,i and m). Taken together, these results indicate that only a small fraction of Bmi1+ tumorigenic cells survived and continued to clonally proliferate independently of each other.

The clonal expansion of Bmi1+ cells was also investigated using *in vitro* three-dimensional organoid culture system (Supplementary Fig. 2a–d). Crypts were collected from *APC*^*min*/+^; *Bmi1*^*CreERT*/+^; *Rosa26*^*rbw*/+^ mice after the Bmi1+ cells in developing tumors were labelled (Supplementary Fig. 2e) and then cultured, showing that most of all crypts formed spherical organoids, differential feature compared with the organoid with budding derived from normal stem cells (Supplementary Fig. 2b–d). Moreover, labelled Bmi1+ cells were detected in the cystic organoid, which clonally expanded from day8 to day 16, as they clonally expanded *in vivo* (Supplementary Fig. 2f–f”). In addition to the proliferation manner, the percentage of the Bmi1+ labelled cells (Supplementary Fig. 2g) was comparable with the *in vivo* data (Fig. 2a).

### Lgr5+cells in proliferating intestine tumors behave as cancer stem cells

**Figure 3:**
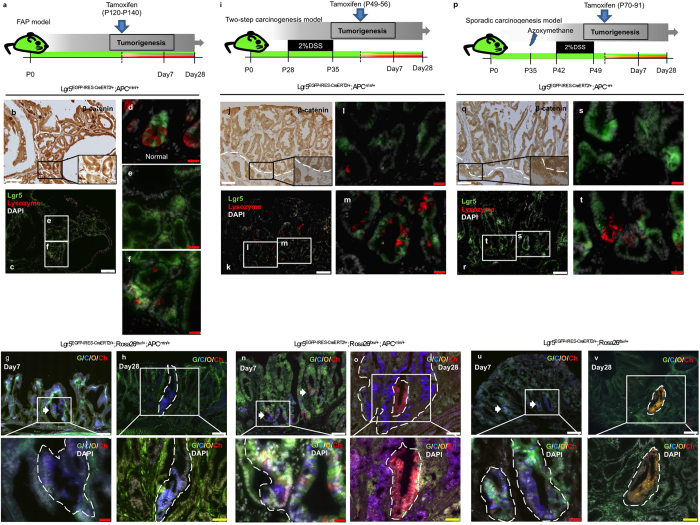
Cell-fate mapping of the Lgr5-positive cells though tumor development in various mouse models. (**a**) Schematic protocol of lineage-tracing of Lgr5-positive cells in tumor-bearing FAP model mice. FAP spontaneously occurs in *APC*^*min*/+^ mice before the age of 140. P, Postnatal day. (i) Schematic protocol of lineage-tracing of Lgr5-positive cells in tumor-bearing two-step carcinogenesis model mice. When *APC*^*min*/+^ mice, which develop FAP, are administrated with 2% DSS from the age of 28 to 35, tumorigenesis is accelerated to generate colon adenoma. P, Postnatal day. (p) Schematic protocol of lineage-tracing of Lgr5-positive cells in tumor-bearing sporadic carcinogenesis model mice. P, Postnatal day. (**b,c,e,f,j–m,q–t**) To investigate the localization of Lgr5+ cells and Paneth cells in the small intestinal tumors and colon tumors, serial sections were prepared from dissected tumors in FAP model mice, two-step carcinogenesis model mice, and sporadic carcinogenesis model mice. One of them was stained for β-catenin to determine the area of tumor mass according to its nuclear localization (**b,j** and **q**) and their serial sections were served for immunostaining of lysozyme together with observation of EGFP expression in order to detect Paneth cells and Lgr5+ cells, respectively (**c,k** and **r**). The area circled with white dotted line represents tumor area, evaluated by nuclear accumulation of β-Catenin. Insets represent higher magnification images of the boxed area. Green; EGFP fluorescence, Red; Lysozyme, White; DAPI. Scale bars, white; 100 μm, yellow; 50 μm, red; 20 μm. (**d**) Representative images of Lgr5+ normal cells in small intestine. (**f,m,t**) Representative images of Lgr5+ tumorigenic cells that coincided with Paneth cells. (**e,l,s**) Representative images of Lgr5+ tumorigenic cells that were independent of Paneth cells. (**g,h,n,o,u,v**) Representative traced images of Lgr5+ cell induced in developing tumors. Tumors were harvested at 7 days after induction (**g,n** and **u**) and 28 days after induction (**h,o** and **v**) in FAP model, two-step carcinogenesis model, and sporadic carcinogenesis model, respectively. G; GFP, C; mCerulean, O; mOrange, Ch; mCherry. Scale bars, white; 100 μm, yellow; 50 μm, red; 20 μm.

**Figure 4:**
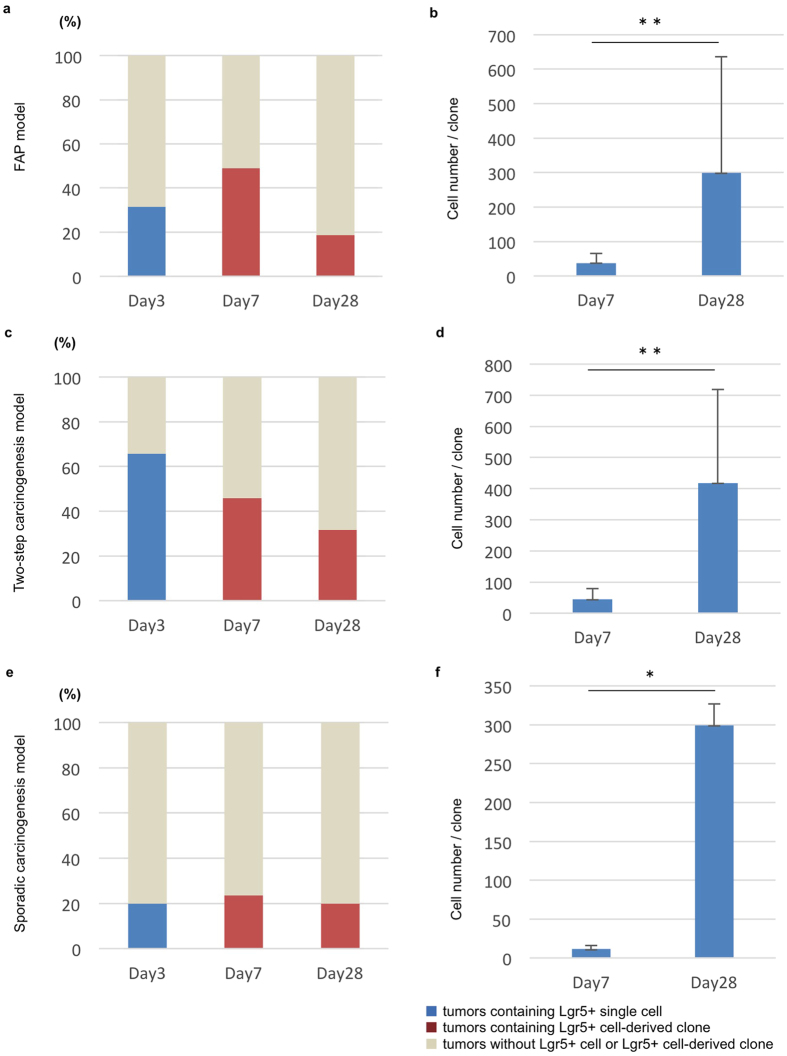
Existence and manner of clonal expansion of Lgr5+ cells in developing small intestinal tumors and colon tumors. (**a,c,e**) The percentage of the developing tumors that contained Lgr5+ positive cells was calculated by observing EGFP using tumor-bearing *Lgr5*^*EGFP-IRES-CreERT2/*^ mice of three models; FAP model (**a**), two-step carcinogenesis model (**c**), and sporadic carcinogenesis model (**e**). To assess the percentage of the tumors containing Lgr5+ cell-derived clone, FAP model, two-step caricinogenesis model, and sporadic carcinogenesis model were set up using *Lgr5*^*EGFP-IRES-CreERT2*/+^*; Rosa26*^*rbw*/+^ mice, followed by tamoxifen induction and chase to trace the lineage of Lgr5+ cell (**a,c,e**): Day7 and Day28. This analysis excluded GFP+ clones because we cannot judge whether the origin of the GFP+ clone is the cell that expressed GFP accompanied by Lgr5 or the Lgr5-negative cell, owing to ubiquitous expression of GFP in *Rosa*^*rbw*^ mice. The number of mice and tumors analyzed were shown in Supplementary Table 3. (**b,d,f**) The number of the cells that comprised each Lgr5+ cell-derived clone was measured, and the average is shown. Cell number per clone at day28 after tamoxifen induction was compared with day7. Error bars indicate standard deviation. **p < 0.01, *p < 0.05. The number of tumors analyzed and the raw data are shown in Supplementary Table 2.

Next, we examined the presence of Lgr5+ tumorigenic cells and their ability to clonally expand in three tumor models using a similar experimental approach. *APC*^*min*/+^; *Lgr5*^*EGFP-IRES-CreERT2*^ used in the FAP model (Fig. 3a) and two step-carcinogenesis model (Fig. 3i), and *Lgr5*^*EGFP-IRES-CreERT2*^ mice used in the sporadic carcinogenesis model (Fig. 3p) were examined for EGFP expression indicative of Lgr5+ cell presence in proliferating tumors (Fig. 3c,e,f,k–m, and r–t). Thus, 31.4%, 65.8%, and 20% of tumors in the FAP, two-step carcinogenesis, and sporadic carcinogenesis models, respectively, contained Lgr5+ cells (Fig. 4a,c,e and Supplementary Table 3). Then, lineage tracing of the Lgr5+ cells was performed using mice carrying the *Rosa26*^*rbw*^allele. At day 7 after tamoxifen induction, a large number of Lgr5+ cell-derived labeled cells were observed in all three models (Figs 3g,n,u and 4a,c, e, and Supplementary Table 3); some clones consisted of cells labeled with the same color, suggesting that they were descendants of a single Lgr5+ cell. At day 28 after induction, a definite number of cells composed each Lgr5+ cell-derived clone (Fig. 3h,o and v), and the cell number per clone was significantly higher than at day 7 (Fig. 4b,d,f and Supplementary Table 2). These results indicate the presence of clonally expanded Lgr5-positive cells in proliferating tumors. Furthermore, proliferating activity varied among Lgr5+ tumorigenic cells as rapidly proliferating cells behaved as cancer-stem-like cells, which clonally expanded. Color patterns of Lgr5-derived neighboring clones were similar to those of Bmi1-derived clones. Ability of Lgr5+ tumorigenic cells to clonally expand was also examined as well as Bmi1+ cells, showing the similar results (Supplementary Fig. 2h–h”,2i).

A previous report showed that Paneth cells were often located adjacent to Lgr5+ adenoma cells, suggesting that they serve as an adenoma stem cell niche^11^, as well the principal cell type of the normal small intestine (Fig. 3d). Although normal colon tissue did not contain Paneth cells, colon adenoma gave rise to adenoma Paneth cells in mice containing Lgr5+ cells with the mutant *Apc* gene^12^. In our study, Paneth cells were detected by immunostaining for lysozyme, whereas tumor area was determined by nuclear localization of β-catenin (Fig. 3b,j and q). FAP mice contained Lgr5+ adenoma cells colocalized with Paneth cells (Fig. 3f) as well as with other cell types (Fig. 3e). Similar heterogeneity was also observed in colon tumors (Fig. 3l,m,s and t), suggesting that our *Apc*^*min*^ and sporadic carcinogenesis models provided the detection of Lgr5+ tumor cells, which did not require niche Paneth cells and were not generated in a previous study based on mice in which tumors are induced by different procedure^11^.

### Lgr5 and Bmi1 play differential roles in tumor formation and progression

To compare the ability of Lgr5- or Bmi1-positive cells to clonally expand at tumor initiation and development, we examined mice injected with tamoxifen before tumorigenesis. Two types of Lgr5+ cell-derived tumors were observed: one contained cells labeled with the same color (Supplementary Fig. 3a–c); the other, with different colors (Supplementary Fig. 3d–f). The former tumors tended to be smaller. These observations led us to hypothesize that Lgr5+ cell-derived tumors were first monoclonal, and then incorporated neighboring clones to generate polyclonal tumors (Supplementary Fig. 4). To test whether there was a correlation between tumor size and clonality, we classified tumors into large (diameter >700 μm) and small (diameter ≤700 μm) (Supplementary Fig. 1). Neither two-step carcinogenesis model mice (Supplementary Fig. 3b–b”, Supplementary Table 4) nor sporadic carcinogenesis model mice (Supplementary Fig. 3c–c”, Supplementary Table 4) developed polyclonal small-size tumors, whereas 50% and 100%, respectively, of them developed polyclonal large-size tumors (Supplementary Table 4, Supplementary Fig. 3e,e’,f and f’). In contrast, Bmi1+ cell-derived tumors were very rare: they were not detected in the two-step carcinogenesis model and only two polyclonal tumors were observed in the sporadic carcinogenesis model (Supplementary Fig. 3h, Supplementary Table 4). Given that Bmi1+ cells were shown to contribute to the clonal expansion in the developing tumors (Fig. 1e,i and m), these data suggest that Bmi1 expression was upregulated during tumor development.

Next, the clonality of Lgr5+ or Bmi1+ cell-derived small intestine tumors was examined using the FAP model, which provided similar results as with colon tumors. Thus, all small-size Lgr5+ cell-derived tumors were monoclonal (Supplementary Fig. 3a–a” and Supplementary Table 4) and the majority (81%) of large-size tumors were polyclonal, whereas Bmi1+ cell-derived small-size tumors were not detected and all large-size tumors were polyclonal (Supplementary Fig. 3g and Supplementary Table 4).

### Colon tumors change from monoclonal to polyclonal during progression

It has long been argued whether tumors are of unicellular or multicellular origin. The monoclonal theory was first proposed based on the analysis of female cancer patients heterozygous for the X-linked glucose-6-phophate dehydrogenase (G-6-PD) locus: a given tumor mass was shown to express enzymes of either type A or B, whereas both A and B were found in normal tissues, suggesting that various types of tumors, including colon carcinomas, are monoclonal^13^. In contrast, there is a report showing that at least 76% of microadenomas in an XO/XY mosaic individual with FAP were polyclonal in origin, as indicated by *in situ* hybridization with Y chromosome probes^14^. Although the evidence remains controversial, our lineage tracing for Bmi1-positive and Lgr5-positive cells during tumor initiation and propagation supports the monoclonal theory (Supplementary Fig. 4). To further substantiate this notion, we performed detailed analysis using ubiquitous multicolor lineage tracing, where all mouse cells were labeled with four fluorescent proteins, and tried to trace cell lineage in the three tumor models. A tumor was distinguished from the surrounding normal tissues by nuclear accumulation of β-catenin^3^ (Supplementary Fig. 1) and was considered monoclonal when all tumor cells expressed one fluorescent protein and polyclonal when they expressed multiple fluorescent proteins.

First, we injected tamoxifen into *APC*^*min*/+^; *Rosa26*^*CreERT2/rbw*^ mice at postnatal day 14 (P14) and DSS P28 to P35 later, in order to analyze tumor clonal composition at the early stage^8^ (Supplementary Fig. 5a). These two-step carcinogenesis models showed that 41.4% of the large-size colon tumors were polyclonal (Supplementary Fig. 5b–b” and Supplementary Table 4), whereas all small-size tumors were monoclonal, which was confirmed by confocal imaging of 70-μm edge-to-edge sections of whole tumor masses (Supplementary Figs 5c–j, 6). The color pattern of polyclonal large-size tumors (Supplementary Fig. 5b”) was similar to that observed in multicolor lineage tracing of Lgr5-positive cells (Supplementary Fig. 3e). Given the existence of large-size monoclonal tumors (58.6%), (Supplementary Table 4 and Supplementary Fig. 7b–b”), these data indicate that tumor clonality in the two-step carcinogenesis models underwent changes consistent with the monoclonal theory. Thus, clonal expansion of tumorigenic cells first gave rise to small-size monoclonal tumors, followed either by further development to large-size monoclonal tumors or by the merge with adjacent monoclonal tumors resulting in a large-size polyclonal tumor (Supplementary Fig. 5b”).

For ubiquitous multicolor lineage-tracing in the sporadic carcinogenesis model, *Rosa26*^*CreERT2/rbw*^ mice were intraperitoneally injected with azoxymethane followed by DSS administration^10^ (Supplementary Fig. 8a). The majority (76.9%) of large-size colon tumors were polyclonal (Supplementary Figs 7c–c”, 8b–b” and Supplementary Table 4), whereas all small-size tumors were monoclonal (Supplementary Figs 8c–m and 9 and Supplementary Table 4). These data on the clonal origin of colon tumors are consistent with those obtained in the two-step carcinogenesis model.

### Small intestine tumors exhibit clonal pattern different from that of colon tumors

In addition to colon tumors, we analyzed small intestine tumors using the FAP model^9^. First, ubiquitous multicolor lineage tracing was performed using *Rosa*^*creERT2/rbw*^; *Apc*^*min*/+^ mice (Supplementary Figs 10a and 11a). Although, similar to colon tumors, most of large-size intestinal tumors (63.2%) were polyclonal (Supplementary Figs 7a–a”, 10c–c”, Supplementary Table 4), not all small-size tumors (92.1%) were monoclonal (Supplementary Figs 10d–k and 11b–g). The polyclonal small-sized tumors were microscopically retained cells^3^ (Supplementary Fig. 11h–h”), presenting a single adenoma-like crypt. Intestinal cancer is considered to start with the invagination of mutated crypt stem cells around the crypt-villus junction and their invasion into the subepithelium^15^. Because a single normal crypt contains multiple cells with a stem-like phenotype^16^, polyclonal small-size tumors may originate as a consequence of the invasion of several mutated stem cells, which then produce different clones. Based on our data, we suggest the following mechanism of tumor progression in the small intestine: original tumors are polyclonal; then, a single Lgr5- or Bmi1-positive cell proliferates and replaces other cells by clonal competition, resulting in the formation of large-size monoclonal tumors. However, when neighboring monoclonal tumors merge, a large-size polyclonal tumor is formed, which is similar to the development of colon tumors. Although our data suggest small intestine tumors exhibit clonal pattern different from that of colon tumors, we have to mention that we cannot exclude the possibility that the polyclonal origin of colon tumors is just not detected because the models used in this study are difficult to detect the very early stage in onset of colon tumors. There seems room for improvement on the model.

### Clonal expansion of cancer stem cell-derived tumors is mediated by crypt fission

Conceptually, the proliferation of intestinal tumors is mediated by crypt fission^12^. Although crypt length was similar in small intestine tumors (FAP) and normal tissues (Supplementary Fig. 12a,c and k), it was shorter than normal in colon tumors, as demonstrated in the two-step and sporadic carcinogenesis models (Supplementary Fig. 12b,d,e and k), suggesting the occurrence of crypt fission during tumor expansion. Proliferation of tumorigenic cells mediated by crypt fission can explain the expansion of adjacent clones without merging with each other.

## Discussion

Our study comprehensively analyzed the contribution of Bmi1+ and Lgr5+ cells to tumor initiation and propagation using multi-color cell-fate mapping. *Rosa*^*rbw*^ mice used in these experiments provided more detailed analysis of tumor clonality compared to previous studies, because more fluorescent reporter genes were used. Regarding the origin in the tumor initiation in small intestine and colon, there have been two opinions, one of which is Bottom-up model^11^,^17^ and the other of which is Top-down model^18^. The Top-down model claims that genetically altered cells in the superficial portions of the mucosae spread laterally and downward to form new crypts that first connect to preexisting normal crypts and eventually replace them^18^, whereas based on the Bottom-up model, crypt fission is essential and initial event when clonal expansion of mutated clones in adenoma occurs^17^. Our observations suggested the Lgr5+ cell located at the crypt base as cell-of-origin of intestine tumors, and the shorter crypts in tumors compared with normal tissues indicated that they had proliferated via crypt fission. Therefore the Bottom-up model seemed to be consistent with our results.

Our observations indicate that not all of Bmi1+ or Lgr5+ tumorigenic cells behave as cancer stem cells. As proposed by Fearon ER and Vogelstein B, colon cancer is considered arising thorough multiple hits of mutation^7^. In addition, several mutations are usually observed in small intestine tumors. Only one characteristic of tumor cells, such as expression of Bmi1 or Lgr5, seem not be sufficient to define the competency of being introduced with multiple mutations. It is therefore suggested that additional markers for cells contributing to clonal expansion in developing tumors need to be identified.

Although our system of multi-color cell-fate mapping is powerful tool to identify cancer stem cells in model mice, it is unfortunately unavailable in human body, because it needs genetically knocked-in construct to induce multi-colors. However, it is much of interest whether Lgr5+ or Bmi1+ cells in human intestine tumors clonally expand. At present, it is difficult to knock-in rainbow construct into patient-derived human tumor cells using, for example, CRISPR/Cas9 technology because the size of the rainbow construct is too large to be knocked-in by such procedure. However, advanced CRISPR/Cas9 technology would let us do that in future. By adding 4-OH tamoxifen into culture medium, multicolor lineage tracing *in vitro* is available. If human cancer cells with rainbow construct would be available, *in vitro* lineage-tracing using these must be helpful to investigate the manner how Bmi1+ - or Lgr5+-cells clonally expand in human intestine tumors.

The relationship between Lgr5+ stem cells, shown in this study to be cells-of-origin for colon carcinoma and small intestine tumors, and Lgr5-expressing tumorigenic cells, which were observed to clonally expand, remains unclear. If these characteristics belong to the same lineage, the initiation and progression of colon carcinoma should be supported by the transition of Lgr5+ stem cells to self-renewing cancer stem cells. However, considering that doublecortin-like kinase 1 (Dclk1) marks stem cells in colon cancer but differentiated cells in normal tissue^19^, it can be suggested that distinct sets of biomarkers exist in tumor-initiating cells and cancer stem cells. Future studies tracing cell fate at different stages of cancer development, i.e., tumor initiation and tumor proliferation, are required to test this hypothesis.

## Materials and Methods

### Animals

C57BL/6J, *APC*^*min*/+^ 9, *Bmi1*^*CreERT/+*^ 1, and *Lgr5*^*GFP-IRES-CreERT2*/+^ 2 mice were purchased from Jackson Laboratories. *Rosa26*^*rbw*/+^ and *Rosa26*^*CreERT2/+*^mice were generated as previously described^4^,^5^,^6^. Mice were bred and maintained at the Kansai Medical University Research Animal Facility in accordance with the Kansai Medical University guidelines. All animal protocols were approved by the Kansai Medical University Walfare Committee.

### Tumor models

*APC*^*min*/+^ mice used as a FAP model^9^ were sacrificed 140 days after birth. To generate a two-step carcinogenesis model, 4-week-old *APC*^*min*/+^ mice were given water containing 2% DSS (MP Biomedicals) for 7 days, left untreated for the following 4 weeks, and then sacrificed^8^. A sporadic carcinogenesis model was created by injecting 35-day-old C57BL/6J mice intraperitoneally with azoxymethane (10 mg/kg body weight; Sigma) and then giving them 2% DSS-containing water for 7 days starting from the age of 42 days^10^; mice were sacrificed at the age of 98 days.

### Tamoxifen induction

To induce CreERT2-mediated multi-color labeling in the FAP and two-step carcinogenesis models, 2-week-old mice were intraperitoneally injected with tamoxifen (Sigma) dissolved in corn oil (Sigma); for the sporadic carcinogenesis model, 34-day-old mice were injected with the drug. To track cell fate after tumorigenesis, mice received tamoxifen at the tumor-bearing stage. *Rosa26*^*CreERT2/+*^, *Lgr5*^*GFP-IRES-CreERT2*/+^, and *Bmi1*^*CreERT*/+^ mice were injected with tamoxifen at the doses of 5, 7, and 9 mg per 40 g of body weight, respectively.

### Tumor detection

To determine the tumor area, immunostaining for β-catenin was performed and the area where the nuclear accumulation of β-catenin in the cells was observed was defined as tumor. Representative images are shown in Supplementary Figs 1 and 10b. In all of the investigations, serial sections were prepared, one of which was served for immunostaining for β-catenin and the other of which was assessed for detecting Lgr5+ or Bmi1+ derived tumors.

### Histological analysis

Animals were anesthetized with isoflurane and small intestinal and colonic tumors with surrounding normal tissues were removed and processed to obtain frozen or paraffin-embedded sections, as previously reported^20^,^21^. For lineage-tracing analysis^4^,^5^,^6^, hematoxylin-eosin (HE) staining, and immunohistochemistry, frozen tissues were cut from edge to edge into 7-μm slices at 70-μm intervals. Cell lineages were traced using fluorescence images of 7-μm sections obtained under an OLYMPUS BX63 (Olympus Corporation, Tokyo, Japan) or BZ-9000 (Keyence Corporation, Osaka Japan) microscopes, and three-dimensional (3D) images were reconstructed from micrographs of 70-μm slices obtained under a Nikon C2 confocal microscope (Nikon Instech) using Nikon or Volocity software (Perkin-Elmer).

### Immunohistochemistry

Immunostaining of paraffin-embedded sections was performed using primary antibodies against mouse anti-β-catenin (clone 14/Beta-catenin, 610514, BD Biosciences) and rabbit anti-GFP (clone D5.1, 2956, Cell Signaling) diluted 1:100. Slides were then incubated with goat anti-mouse or anti-rabbit Histofine simple stain immuno-enzyme polymers (MAX-PO (M), 414322, and MAX-PO (R), 414341, respectively, Nichirei Bioscience Inc.), and staining was developed using metal-enhanced 3,3′-diaminobenzidine (DAB). Slides were counterstained with hematoxylin and observed under an OLYMPUS BX41 microscope (Olympus Corporation).

### Tumor classification

Small intestinal neoplasms were classified as small-sized (≤0.7 mm × 0.7 mm) and large-sized (>0.7 mm × 0.7 mm) tumors. Microscopically identified cell groups with nuclear accumulation of β-catenin previously defined as single adenomatous crypts^3^ (Supplementary Fig. 1a) were categorized as small-size tumors. Microadenomas previously defined as lesions that could be microscopically detected without β-catenin staining and not exceeding 2 mm × 2 mm in size^3^ (Supplementary Fig. 1b,c) were classified into small-size or large-size tumors as described above. Adenomas defined as tumors larger than 2 mm × 2 mm^3^ (Supplementary Fig. 1d) were automatically categorized as large-size tumors. Colon tumors were classified using the same criteria.

### Organoid culture

Adenomas were collected from the intestine of *APC*^*min*/+^ mouse and were flushed several times in cold PBS containing 500 μm dithiothreitol (DTT) using transfer pipette. The adenomas were then incubated in 10 ml of chelating buffer at 4 °C with constant stirring for 20 min. The chelating buffer (pH 7.3) contained 27 mM trisodium citrate, 5 mM Na_2_HPO_4_, 94 mM NaCl, 8 mM KH_2_PO_4_, 1.5 mM KCl, 0.5 mM DTT, 55 mM D-sorbitol and 44 mM sucrose^22^. The adenomas were then transferred to 5 ml of fresh cold chelating buffer and vigorously shaken by hand (20 inversions). The chelating buffer was replaced with 5 ml of fresh cold chelating buffer. This procedure was repeated 4–5 times to remove villi. The adenomas were again incubated in 10 ml of chelating buffer at 4 °C with constant stirring for 10 min and crypts were released from the adenomas into the chelating buffer by vigorous shaking by hand (20 inversions). Crypts collected from adenomas or normal intestine were cultured in Matrigel (Becton Dickinson Biosciences, San Jose, CA), in the presence of cytokines^23^. Briefly, the crypts were suspended in Matrigel (0.3–0.5 × 10^4^ cells/50 μl of Matrigel) and plated 24-well plate (triplicate). Then 0.75 ml of advanced DMED/F-12 medium supplemented with N-2, B-27, N-acethyl cysteine, Glutamax (Invitrogen) and cytokines (rmEGF: 50 ng/ml, rmnoggin: 100 ng/ml, rhR-spondin1-hFc: 1000 ng/ml) was added into Matrigel. The EGF and noggin were purchased from Peprotech. The R-spondin1-hFc containing a C-terminal of human IgG was produced in our laboratory. The cDNA of rhR-spondin1 was kindly donated by Kyowa Hakko Kirin (Tokyo, Japan). Y-27632 (10 μM, Sigma) was also added to the culture medium. Every 2 or 4 days, all the culture medium in the wells was replaced with fresh medium.

### Statistical analyses

Crypt length was assessed in HE-stained sections of the normal small intestine and colon, and in β-catenin-stained sections of tissues from FAP, two-step carcinogenesis, and sporadic carcinogenesis model mice. The representative images to explain how we measured the length of crypt in normal tissues and tumors are shown in Supplementary Fig. 12f–j. Sections from three mice in each group were observed and analyzed by Student’s *t*-test. The numbers of cells in the Bmi1+ or Lgr5+ clone lineages of developing tumors were compared at days 7 and 28 after tamoxifen induction using Welch’s test.

### Ethics statement

All animal experiments were performed in accordance with the Kansai Medical University guidelines and approved by the Kansai Medical University Animal Experiment Committee.

## Additional Information

**How to cite this article**: Yanai, H. *et al*. Intestinal cancer stem cells marked by Bmi1 or Lgr5 expression contribute to tumor propagation via clonal expansion. *Sci. Rep.* **7**, 41838; doi: 10.1038/srep41838 (2017).

**Publisher's note:** Springer Nature remains neutral with regard to jurisdictional claims in published maps and institutional affiliations.

## Supplementary Material

Supplementary Figures
