# Supplementary material for: Intestinal cancer stem cells marked by Bmi1 or Lgr5 expression contribute to tumor propagation via clonal expansion
Source: Sci Rep. 2017 Feb 8;7:41838. doi: 10.1038/srep41838 (PMC5296906; doi:10.1038/srep41838)
Supplement: Supplementary Figures [file srep41838-s1.docx]

**Intestinal cancer stem cells marked by Bmi1 or Lgr5 expression contribute to tumor propagation via clonal expansion.**

^1.2^Hirotsugu Yanai, ^1^Naho Atsumi, ^1, 3^Toshihiro Tanaka, ^1,3^Naohiro Nakamura, ^1,4^Yoshihiro Komai, ^1,5^Taichi Omachi, ^1^Kiyomichi Tanaka, ^1^Kazuhiko Ishigaki, ^1^Kazuho Saiga, ^1,4^Haruyuki Ohsugi, ^1^Yoko Tokuyama, ^1^Yuki Imahashi, ^1, 6^Shuichi Ohe, ^1^Hiroko Hisha, ^1^Naoko Yoshida, ^1^Keiki Kumano, ^2^Masanori Kon and ^1^Hiroo Ueno*

^1^Department of Stem Cell Pathology, Kansai Medical University, 2-5-1 Shin-machi, Hirakata, Osaka 573-1010, Japan

^2^Department of Surgery, Kansai Medical University, 2-5-1 Shin-machi, Hirakata, Osaka 573-1010, Japan

^3^Third Department of Internal Medicine, Kansai Medical University, 2-5-1 Shin-machi, Hirakata, Osaka 573-1010, Japan

^4^Department of Urology and Andrology, Kansai Medical University, 2-5-1 Shin-machi, Hirakata, Osaka 573-1010, Japan

^5^Department of Pediatrics, Kansai Medical University, 2-5-1 Shin-machi, Hirakata, Osaka 573-1010, Japan

^6^Department of Dermatology, Kansai Medical University, 2-5-1 Shin-machi, Hirakata, Osaka 573-1010, Japan

*Correspondence and requests for materials should be addressed to H.U. (hueno@hirakata.kmu.ac.jp)

**Supplementary Materials**

**
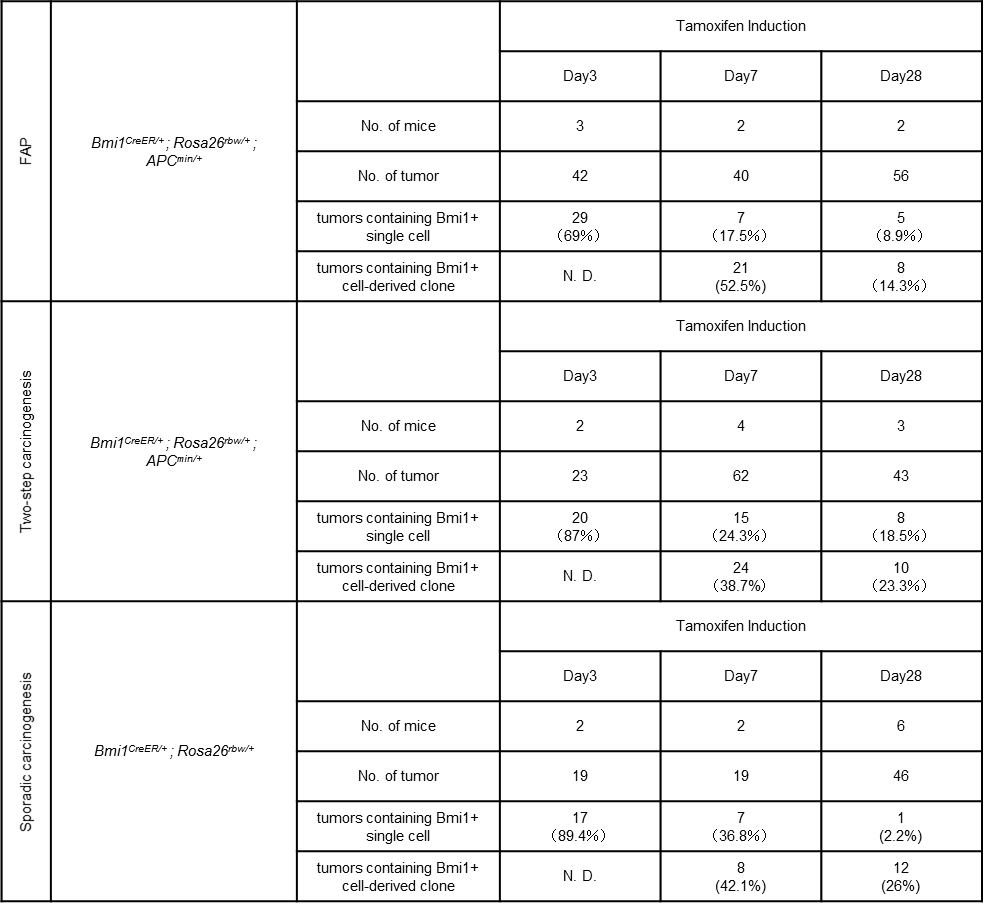
**

**Supplementary Table 1 Existence and manner of clonal expansion of Bmi1+ cells in developing small intestinal tumors and colon tumors were analyzed using lineage tracing of Bmi1+ cells.**

To assess the existence of Bmi1+ cells in developing tumor and the percentage of the tumors containing Bmi1+ cell-derived clone, FAP model, two-step caricinogenesis model, and sporadic carcinogenesis model were set up using *Bmi1^CreERT/+^ ; Rosa26^rbw/+^* mice. The percentage of the developing tumors that contained Bmi1+ positive cells was calculated by tamoxifen induction of tumor-bearing mice followed by 3-day chase and observation of the cells with rainbow color; mCerulean, mOrange, and mCherry (Day3). To evaluate the percentage of the tumors containing Bmi1+ cell-derived clone, tumor-bearing mice of three models were injected with tamoxifen and clones with rainbow color were examined at 7days or 28days after induction (Day7, and Day28). At the same time, tumors that contained rainbow-colored single cell, which was suggested to express Bmi1 when tamoxifen induction but had not divided, were categorized as “tumors containing Bmi1+ single cell”. At day3 after induction, because colored Bmi1+ cell had neither divided nor produced clones yet, “tumors containing Bmi1+ cell-derived clone” were not determined (N.D.).

**
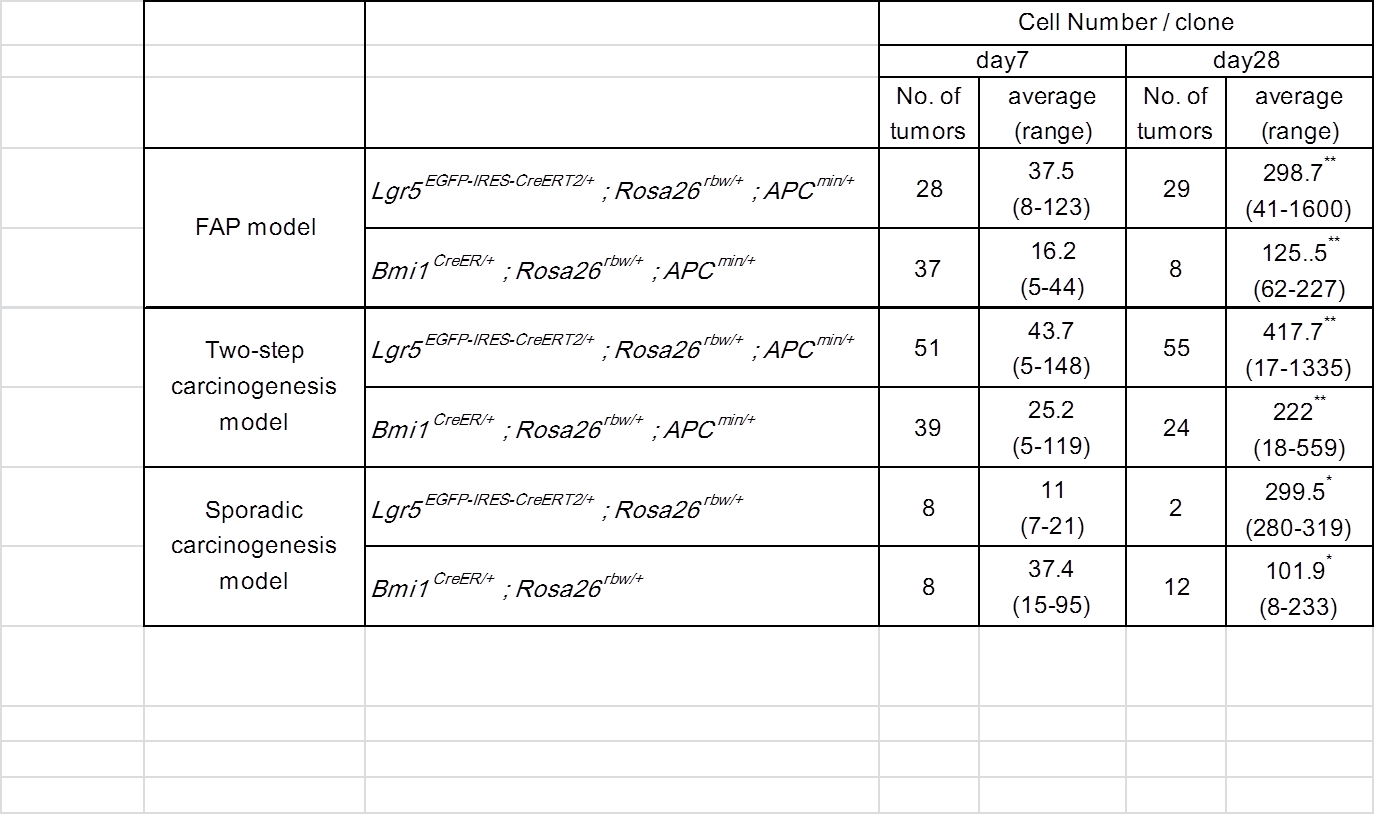
**

**Supplementary Table 2 The size of Bmi+ cell- or Lgr5+ cell-derived clones increases thorough tumor progression in all three models.**

The number of the cells that comprised each clone was measured, and the average and range is shown. Cell number per clone at day28 after tamoxifen induction was compared with day7. **; p<0.01, *; p<0.05

|  | | **GFP+ tumors / total tumor number** | **tumors containing Lgr5+ cell-derived clone / total tumor number** | |
| --- | --- | --- | --- | --- |
| **FAP** |  | **Tamoxifen Induction** | | |
|  |  | **No induction**  ***Lgr5^EGFP-IRES-CreERT2/+ ;^ APC^min/+^*** | **Day7**  ***Lgr5^EGFP-IRES-CreERT2/+^ ; Rosa26^rbw/+^ ; APC^min/+^*** | **Day28**  ***Lgr5^EGFP-IRES-CreERT2/+^ ; Rosa26^rbw/+^ ; APC^min/+^*** |
|  | **No. of mice** | **3** | **3** | **3** |
|  |  | **17 / 54**  **(31.4%)** | **23 / 47**  **(48.9%)** | **13 / 70**  **(18.6%)** |
| **Two-step carcinogenesis** |  | **Tamoxifen Induction** | | |
|  |  | **No induction**  ***Lgr5^EGFP-IRES-CreERT2/+ ;^ APC^min/+^*** | **Day7**  ***Lgr5^EGFP-IRES-CreERT2/+^ ; Rosa26^rbw/+^ ; APC^min/+^*** | **Day28**  ***Lgr5^EGFP-IRES-CreERT2/+^ ; Rosa26^rbw/+^ ; APC^min/+^*** |
|  | **No. of mice** | **3** | **2** | **2** |
|  |  | **27 / 41**  **(65.8%)** | **18 / 39**  **(46%)** | **19 / 60**  **(31.7%)** |
| **Sporadic carcinogenesis** |  | **Tamoxifen Induction** | | |
|  |  | **No induction**  ***Lgr5^EGFP-IRES-CreERT2^*** | **Day7**  ***Lgr5^EGFP-IRES-CreERT2/+^ ; Rosa26^rbw/+^*** | **Day28**  ***Lgr5^EGFP-IRES-CreERT2/+^ ; Rosa26^rbw/+^*** |
|  | **No. of mice** | **2** | **2** | **2** |
|  |  | **2 / 10**  **(20%)** | **4 / 17**  **(23.5%)** | **2 / 10**  **(20%)** |

**Supplementary Table 3 Existence and manner of clonal expansion of Lgr5+ cells in developing small intestinal tumors and colon tumors were analyzed using lineage tracing of Lgr5+ cells.**

The percentage of the developing tumors that contained Lgr5+ positive cells was calculated by observing EGFP using tumor-bearing *Lgr5^EGFP-IRES-CreERT2/^* mice of three models; FAP model, two-step carcinogenesis model, and sporadic carcinogenesis model (No induction). To assess the percentage of the tumors containing Lgr5+ cell-derived clone, FAP model, two-step caricinogenesis model, and sporadic carcinogenesis model were set up using *Lgr5^EGFP-IRES-CreERT2/+^ ; Rosa26^rbw/+^* mice, followed by tamoxifen induction and chase to trace the lineage of Lgr5+ cell (Day7 and Day28). This analysis excluded GFP+ clones because we cannot judge whether the origin of the GFP+ clone is the cell that expressed GFP accompanied by Lgr5 or the Lgr5-negative cell, owing to ubiquitous expression of GFP in *Rosa^rbw^* mice.

|  | | **No. of mice** | **tumor size ≦700μm** | | **tumor size＞700μm** | |
| --- | --- | --- | --- | --- | --- | --- |
|  |  |  | **polyclonal** | **monoclonal** | **polyclonal** | **monoclonal** |
| **FAP** | ***Rosa26^rbw/CreERT2^; APC^min/+^*** | **3** | **3**  **(7.9%)** | **35**  **(92.1%)** | **36**  **(63.2%)** | **21**  **(36.8%)** |
|  | ***Lgr5^EGFP-IRES-CreERT2/+^ ; Rosa26^rbw/+^ ; APC^min/+^*** | **3** | **0** | **9**  **(100%)** | **34**  **(81%)** | **8**  **(9%)** |
|  | ***Bmi1^CreERT/+^ ; Rosa26^rbw/+^ ; APC^min/+^*** | **2** | **0** | **0** | **9**  **(100%)** | **0** |
| **Two-step carcinogenesis** | ***Rosa26^rbw/CreERT2^ ; APC^min/+^*** | **2** | **0** | **10**  **(100%)** | **12**  **(41.4%)** | **17**  **(58.6%)** |
|  | ***Lgr5^EGFP-IRES-CreERT2/+^ ; Rosa26^rbw/+^ ; APC^min/+^*** | **3** | **0** | **6**  **(100%)** | **7**  **(50%)** | **7**  **(50%)** |
|  | ***Bmi1^CreERT/+^ ; Rosa26^rbw/+^ ; APC^min/+^*** | **2** | **0** | **0** | **0** | **0** |
| **Sporadic carcinogenesis** | ***Rosa26^rbw/CreERT2^*** | **3** | **0** | **9**  **(100%)** | **10**  **(76.9%)** | **3**  **(23.1%)** |
|  | ***Lgr5^EGFP-IRES-CreERT2/+^ ; Rosa26^rbw/+^*** | **3** | **0** | **1**  **(100%)** | **12**  **(100%)** | **0** |
|  | ***Bmi1^CreERT/+^ ; Rosa26^rbw/+^*** | **2** | **0** | **0** | **2**  **(100%)** | **0** |

**Supplementary Table 4 Clonality of small intestinal tumors and colon tumors was analyzed according to their size.**

Clonality of tumor was evaluated by ubiquitous multicolor lineage tracing (*Rosa26^CreERT2/rbw^* mice), or fate-mapping of Lgr5+ cell (*Lgr5^EGFP-IRES-CreERT2/+^ ; Rosa26^rbw/+^) or* Bmi1+ cell *(Bmi1^CreERT/+^ ; Rosa26^rbw/+^ mice)* in FAP model mice, two-sporadic carcinogenesis model mice, and sporadic carcinogenesis model mice. When the tumors that occurred in *Lgr5^EGFP-IRES-CreERT2/+^ ; Rosa26^rbw/+^* mice *and Bmi1^CreERT/+^ ; Rosa26^rbw/+^* mice were analyzed, GFP+ clones were excluded from examination, because they cannot be judged whether they were derived of the cell that had expressed Lgr5 or Bmi1.

**
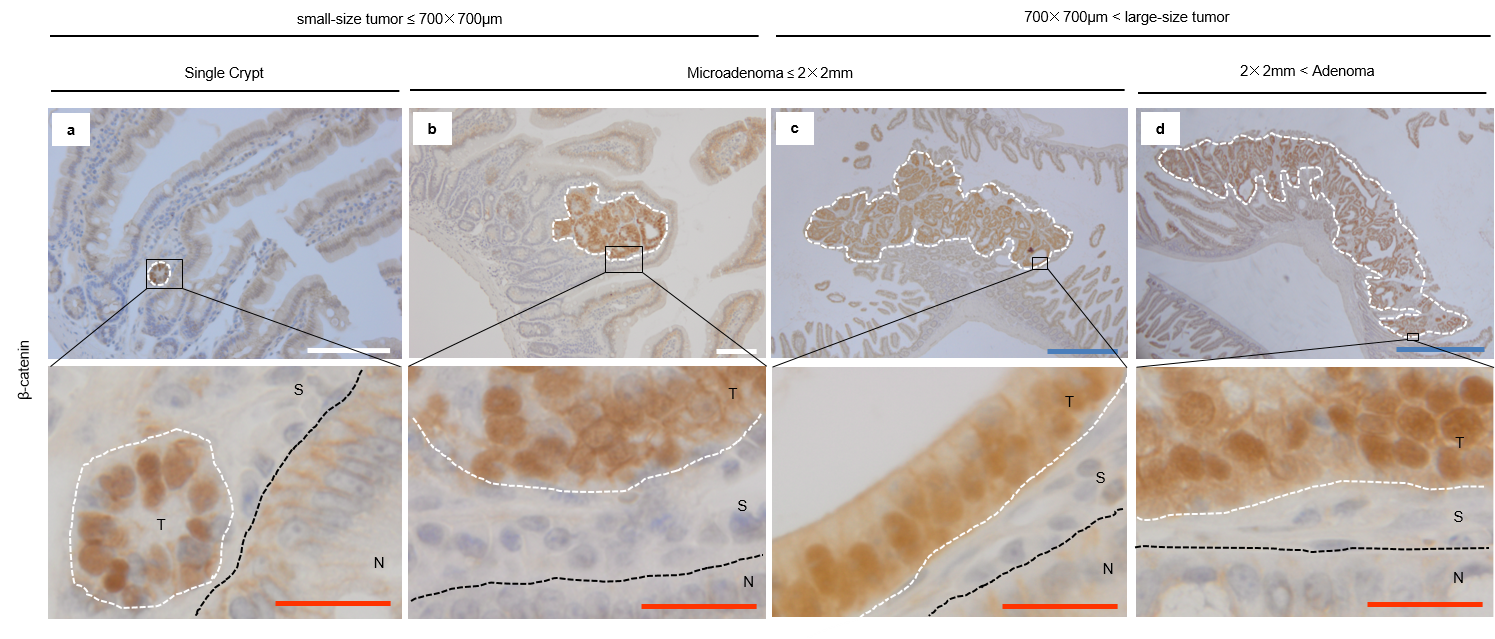
**

**Supplementary Figure 1 Tumor area decided by nuclear accumulation of β-catenin and classification of tumors according to their size.**

T, tumor area; S, Stromal area; N, Normal area. Scale bar, blue; 500μm, white; 100μm, red; 20μm.

**
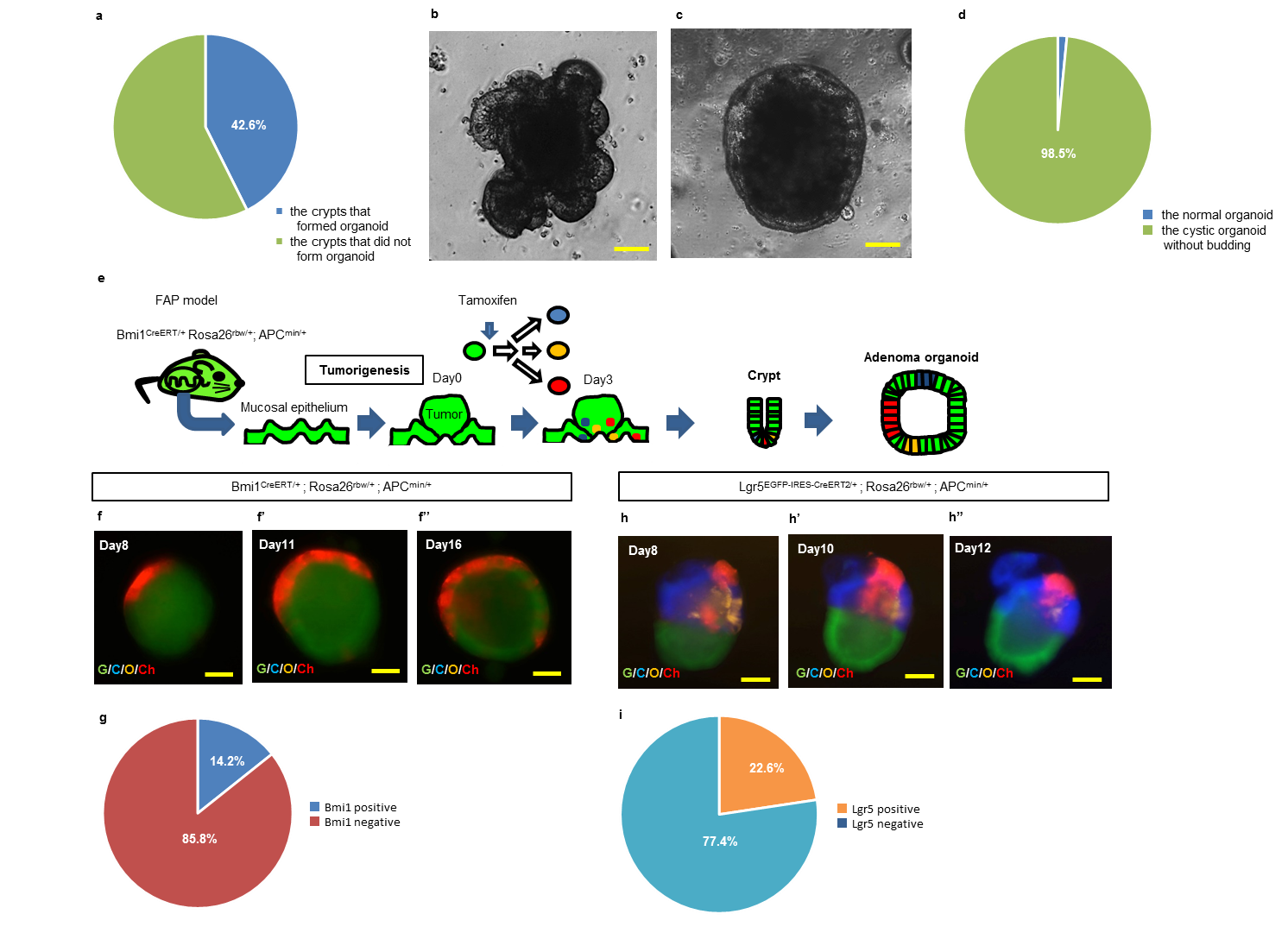
**

**Supplementary Figure 2 Organoid culture using tumorigenic crypts harvested from FAP model mice recapitulated the clonal expansion of Bmi1+ cells and Lgr5+ cells *in vitro*.**

(a) The 43.3% (1169/2700) of crypts harvested from *APC^min/+^* mice formed organoids, whereas the rest of crypts failed to form organoids and disappeared thorough differentiation.

(b) Representative image of organoid with crypt-like structures penetrating toward outside. This type of organoid with budding was considered as derived from normal stem cells. Scale bar, 50μm.

(c) Representative image of spherical organoid, which was considered as derived from tumorigenic cells. Scale bar, 50μm.

(d) The ratio of the cystic organoids without budding was 98.5% (1151/1169), suggesting that almost all of the organoids were derived from tumorigenic crypts.

(e) Schematic protocol of the CreERT2-mediatid multi-colour labeling in FAP model mice, followed by organoid culture *in vitro*. The case when Bmi1-positive cells in developing tumors were labelled using *Bmi1^CreERT/+^* ; *Rosa26^rbw/+^ ; APC^min/+^* mice is shown.

(f-f”) Tumor organoid that contained the clone derived from labelled Bmi1+ tumorigenic cell (mCherry). The organoids containing labelled cells were marked at day8 and the images of the same organoids were taken every day, showing that the size of Bmi1+ cell-derived clone increased from day 8 to day 18. Scale bar, 50μm.

(g) Of the organoids derived from *Bmi1^CreERT/+^* ; *Rosa26^rbw/+^ ; APC^min/+^* mice, the percentage of those containing labelled Bmi1+ cells was 14.2% (8/56)

(h-h”) Tumor organoid that contained the several clones derived from labelled Lgr5+ tumorigenic cells (mCerulean, mOrange, mCherry). The organoids containing labelled cells were marked at day 8 and the images of the same organoids were taken every day, showing that the size of some Lgr5+ cell-derived clones increased from day8 to day 12 (mCerulean), whereas there existed Lgr5+ cell-derived clones that did not grow or seemed to degenerate, which were suggested to derived from relatively differentiated Lgr5+ cells. Scale bar, 50μm.

(i) Of the organoids derived from *Lgr5^EGFP-IRES-CreERT2/+^* ; *Rosa26^rbw/+^ ; APC^min/+^* mice, the percentage of those containing labelled Lgr5+ cells was 22.6% (19/84)

**
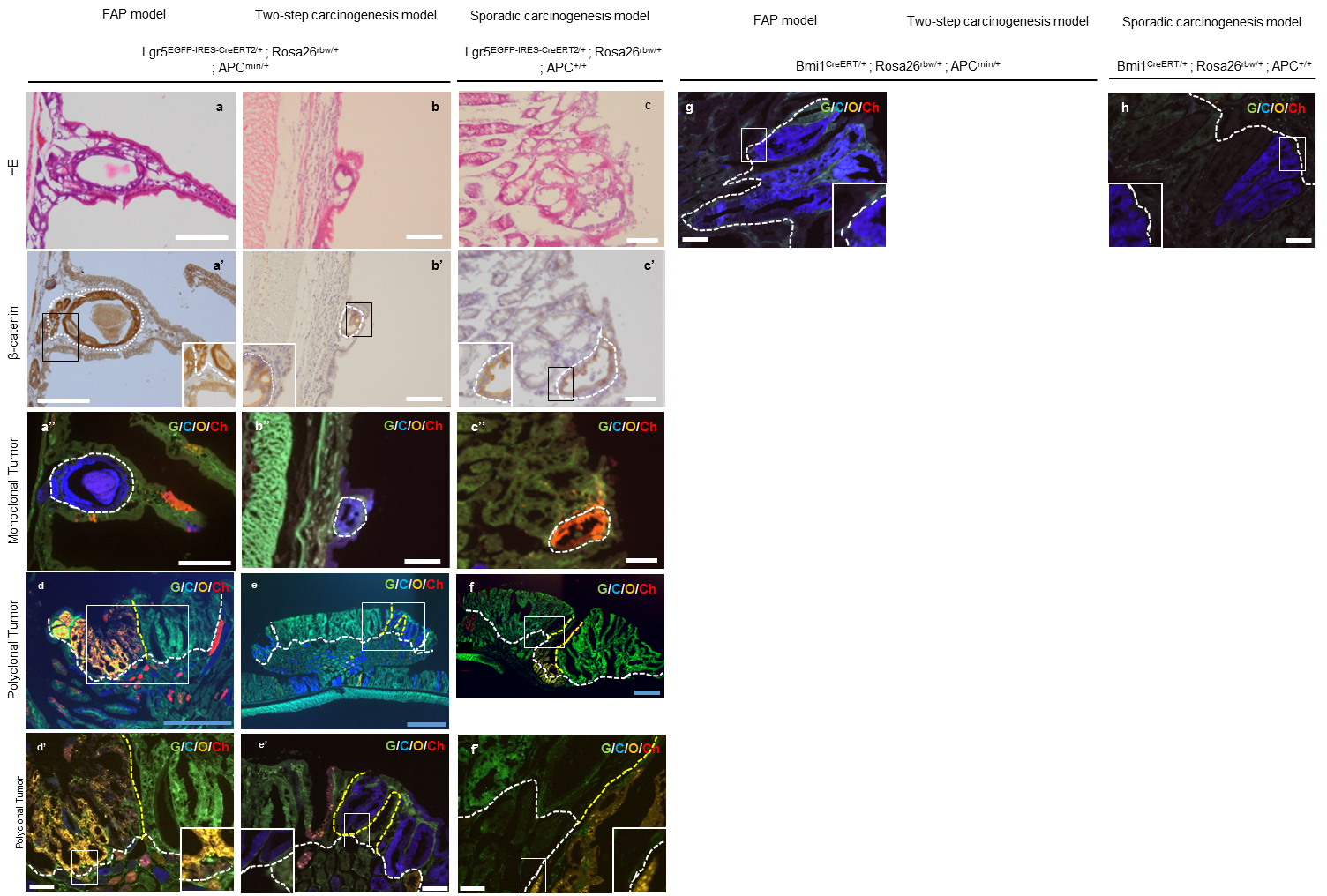
**

**Supplementary Figure 3 The clonal composition changed from monoclonal to polyclonal in three models of intestinal tumors.**

(a, b, c) Representative images of HE staining (a, b, c), β-Catenin (brown) staining (a’, b’, c’) and fluorescent images (a’’, b”, c”) of monoclonal tumors observed in FAP mouse model using *Lgr5^EGFP-IRES-CreERT2/+^ ; Rosa26^rbw/+^ ; APC^min/+^* mice, two-step carcinogenesis model using *Lgr5^EGFP-IRES-CreERT2/+^ ; Rosa26^rbw/+^ ; APC^min/+^* mice, and sporadic carcinogenesis mouse model using *Lgr5^EGFP-IRES-CreERT2/+^ ; Rosa26^rbw/+^ ; APC^+/+^* mice, respectively. Red, Blue and yellow cells represent mCherry-, mCerulean-, and mOrange-labeled cells induced by tamoxifen in *Rosa26^CreERT2/rbw^* mice, respectively.

(d-f) Representative fluorescent images of polyclonal Lgr5+ cell-derived polyclonal tumors observed in FAP mouse model (d), two-step carcinogenesis model (e), and sporadic carcinogenesis mouse model (f), respectively. White dashed lines indicate the boundary between tumor and surrounding normal tissue. Yellow dashed lines indicate the boundary between different tumor clones. Scale bar, 500μm.

(d’-f’) Higher power field of boxed areas in correspondent images (d, e, and f) are shown (d’, e’, and f’, respectively). Inlet images show representative boundary images between tumors and normal tissues. White dashed lines indicate the boundary between tumor and surrounding normal tissue. Yellow dashed lines indicate the boundary between different tumor clones. Scale bar, 100μm.

(g, h) Representative fluorescent images of polyclonal Bmi1+ cell-derived tumors observed in FAP mouse model (g), and sporadic carcinogenesis mouse model (h), respectively. Red, Blue and yellow cells represent mCherry-, mCerulean-, and mOrange-labeled cell

**
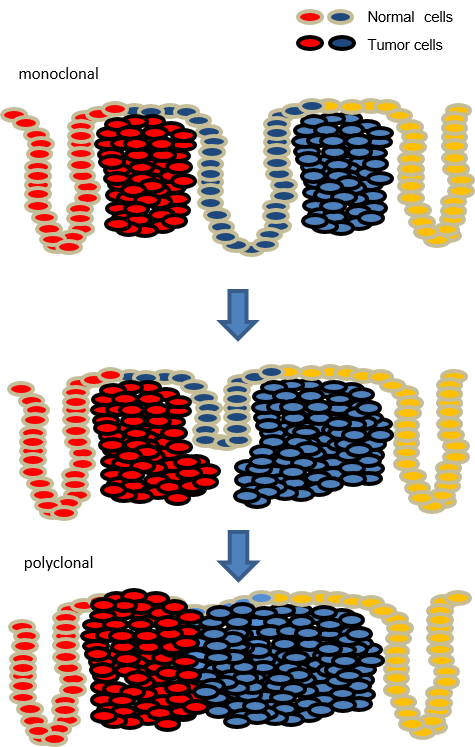
**

**Supplementary Figure 4 Adjacent small-sized monoclonal tumors, where Lgr5+ cells or Bmi1+ cells clonally expand, incorporate to form polyclonal large-sized tumor.**

Schematic model of the development of small intestinal adenoma and colon adenocarcinoma. In earlier stage of tumor formation, each small-sized tumor consists of the cells labeled by the same color suggesting that they are monoclonal. When adjacent developing monoclonal tumors are incorporated with each other, large-sized polyclonal tumor is formed, which consists of clearly different segments with different colors.


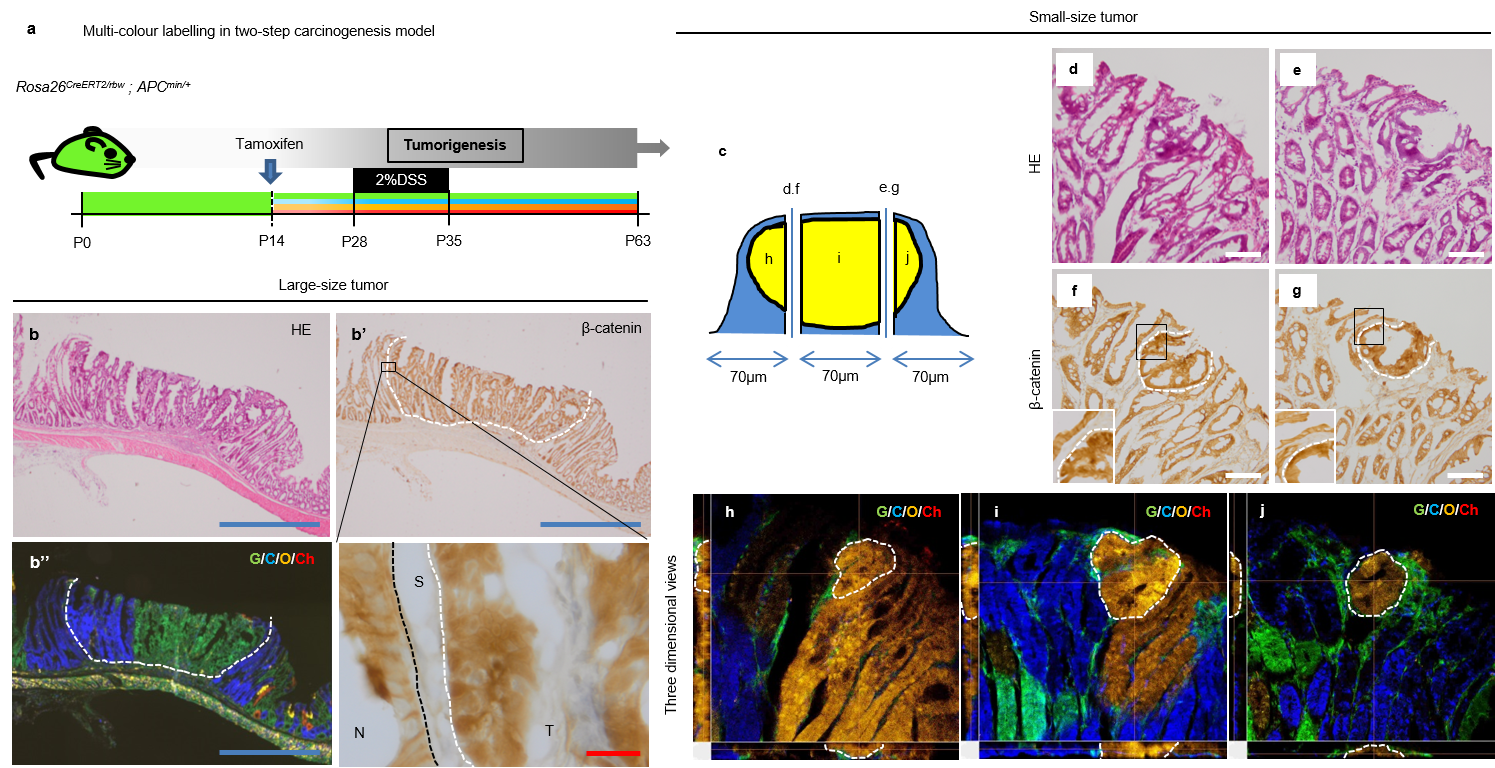


**Supplementary Figure 5 Lineage tracing using tamoxifen-inducible multi-colour labeling system to track the cell-fate in two-step carcinogenesis mouse model.**

(a) Schematic protocol of the CreERT2-mediatid ubiquitous multi-colour labeling in two-step carcinogenesis model using *Rosa26^CreERT2/rbw^ ; APC^min/+^* mice treated with 2% DSS. *Rosa26^CreERT2/rbw^* line was used for the purpose of ubiquitous multicolor lineage tracing. When this mouse, all the cells of which express GFP, received tamoxifen at the age of 14, CreERT2-mediated recombination is occurred in ubiquitous cells, leading to GFP deletion and random expression of mCerulean, mOrange or mCherry. 63-day-old mice were analyzed. P, Postnatal day.

(b) Representative HE staining (b) and fluorescent image (b’’) of large-sized tumor are shown. G, GFP; C, mCerulean; O, mOrange; Ch, mCherry. Representative β-Catenin (brown) staining (b’) and a high-power view of the boxed area (lower-right) are shown. White dotted line and black dotted line represent the border between tumor and stroma, and the border between normal and stroma, respectively. T, tumor; S, Stroma; N, Normal. Scale bar, blue; 500μm, red; 20μm.

(c) Schematic representation of the slice preparation from small-size tumor for HE staining (d, e), immunostaining of β-Catenin (f, g) and three-dimensional analysis of traced cell-lineage (h-j). Yellow-colored area represents the tumor area consisting of the cells derived of a single mOrange-labeled cell; blue area represents surrounding normal tissues consisting of the cells derived of a single mCerulean-labeled cell.

(d, e) Representative images of HE staining using two different slices prepared from the single small-size tumor at the interval of 70μm. Scale bar, 100μm.

(f, g) Representative images of β-Catenin (brown) staining using two different slices prepared from the single small-size tumor at the interval of 70μm. The area circled with white dotted line represents tumor area, evaluated by nuclear accumulation of β-Catenin. Insets represent higher magnification images of the correspondent boxed area. Scale bar, 100μm.

(h-j) Representative three-dimensional images of the different parts of the single small-size tumor, reconstructed from the pictures taken using a confocal microscopy. G; GFP, C; mCerulean, O; mOrange, Ch; mCherry.


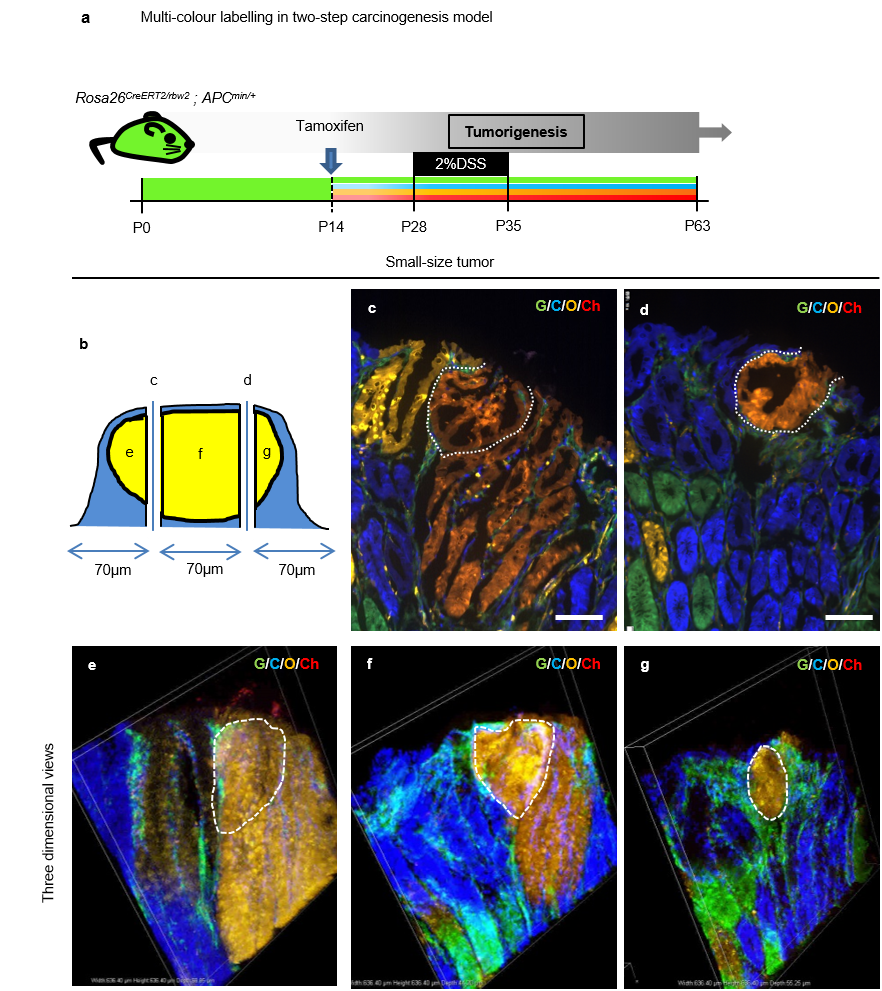


**Supplementary Figure 6 Monoclonal adenoma that occurred in two-step carcinogenesis mouse model was analyzed using multi-colour labeling method.**

(a) Schematic protocol of the CreERT2-mediatid ubiquitous multi-colour labeling in two-step carcinogenesis model using *Rosa26 ^CreERT2/rbw^ ; APC^min/+^* mice. P, Postnatal day.

(b) Schematic representation of the slice preparation from small-size tumor for fluorescent image (c, d) and three-dimensional analysis (e-g) of traced cell-lineage. Yellow-colored area represents the tumor area consisting of the cells derived of a single mOrange-labeled cell; blue area represents surrounding normal tissues consisting of the cells derived of a single mCerulean-labeled cell.

(c,d) Representative fluorescent images the different parts of the single small-size tumor are shown. Scale bar, 100μm.

(e-g) Representative three-dimensional images of the different parts of the single small-size tumor, reconstructed from the pictures taken using a confocal microscopy. G; GFP, C; mCerulean, O; mOrange, Ch; mCherry.


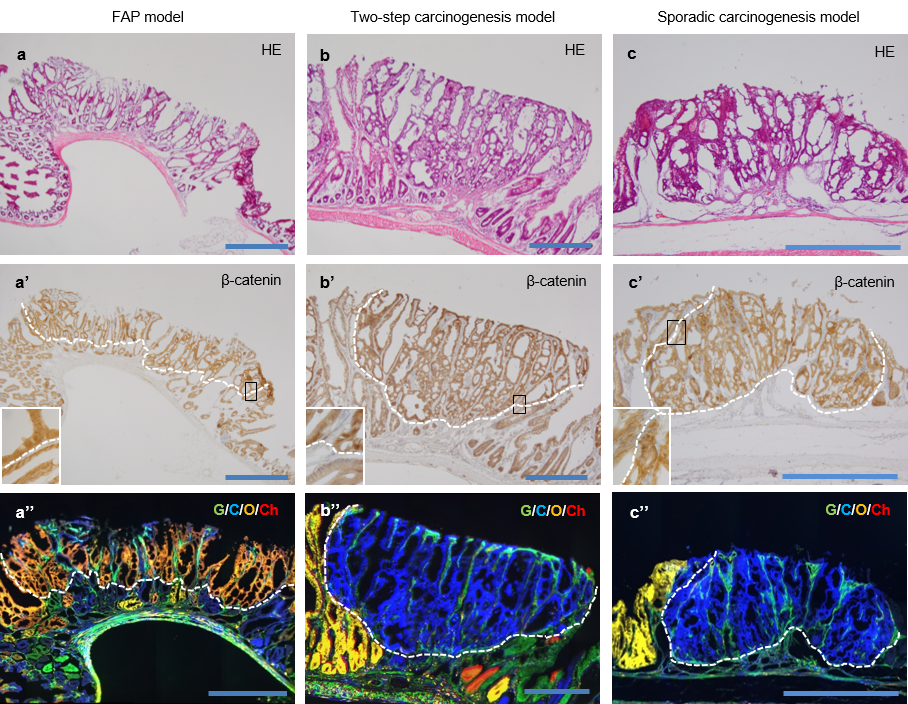


**Supplementary Figure 7 Monoclonal large-size tumors that occurred in FAP mouse model, two-step carcinogenesis mouse model, and sporadic carcinogenesis mouse model analyzed using multi-colour labeling method.**

(a, b, c) Representative images of HE staining using slices prepared from the large-size tumor in FAP model (a), two-step carcinogenesis model (b), and sporadic carcinogenesis model (c). Scale bar, 500μm.

(a’, b’, c’) Representative images of β-Catenin (brown) staining using slices prepared from the large-size tumor in FAP model (a’), two-step carcinogenesis model (b’), and sporadic carcinogenesis model (c’). The area circled with white dotted line represents tumor area, evaluated by nuclear accumulation of β-Catenin. Insets represent higher magnification images of the correspondent boxed area. Scale bar, 500μm.

(a’’, b’’, c’’) Representative fluorescent images of the large-size tumor showed that there existed monoclonal large size tumor in FAP model (a’’), two-step carcinogenesis model (b’’) and sporadic carcinogenesis model (c’’). Because the blue or green colored cells (a’’), or green colored cells (b’’ and c’’) in tumor mass were confirmed not to be epithelial cells (Data not shown), the tumors were judged as monoclonal. G; GFP, C; mCerulean, O; mOrange, Ch; mCherry. Scale bar, 500μm.


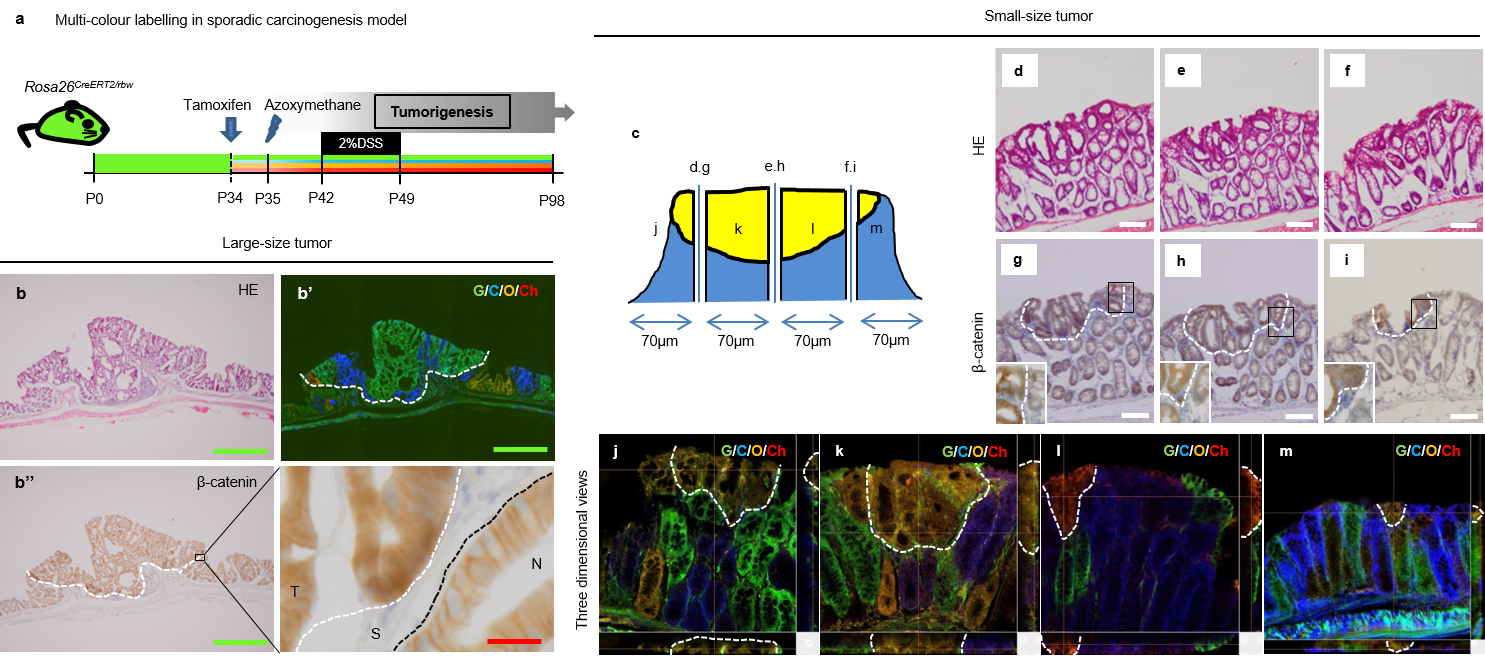


**Supplementary Figure 8 Clonal analysis of adenocarcinoma occurring in sporadic carcinogenesis mouse model by multi-colour lineage tracing.**

(a) Schematic protocol of the CreERT2-mediatid ubiquitous multi-colour labeling in sporadic carcinogenesis model using *Rosa26^CreERT2/rbw^ ; APC^+/+^* mice. When the mice, all the cells of which express GFP, received tamoxifen at the age of 34, CreERT2-mediated recombination is occurred in ubiquitous cells, leading to GFP deletion and random expression of mCerulean, mOrange or mCherry. Single administration of azoxymethane at the age of 35 initiates tumorigenesis, followed by 2% DSS treatment from the age of 42 to 49, which promotes tumorigenesis to generate sporadic colon adenocarcinoma. 98-day-old mice were analyzed. P, Postnatal day.

(b) Representative HE staining (b) and fluorescent image (b’) of large-size tumor are shown. G; GFP, C; mCerulean, O; mOrange, Ch; mCherry. Representative β-Catenin (brown) staining (b’’) and a high-power view of the boxed area (lower-right) are shown. White dotted line and black dotted line represent the border between tumor and stroma, and the border between normal epithelium and stroma, respectively. T, tumor; S, Stroma; N, Normal. Scale bar (green, shown in upper-left, lower-left and upper-right), 1mm; (red, shown in lower-right), 20μm.

(c) Schematic representation of the slice preparation from small-size tumor for HE staining (d-f), immunostaining of β-Catenin (g-i) and three-dimensional analysis of traced cell-lineage (j-m). Yellow-colored area represents the tumor area consisting of the cells derived of a single mOrange-labeled cell; blue area represents surrounding normal tissues consisting of the cells derived of a single mCerulean-labeled cell.

(d-f) Representative images of HE staining using two different slices prepared from the single small-size tumor at the interval of 70μm. Scale bar, 100μm.

(g-i) Representative images of β-Catenin (brown) staining using two different slices prepared from the single small-size tumor at the interval of 70μm. The area circled with white dotted line represents tumor area, evaluated by nuclear accumulation of β-Catenin. Insets represent higher magnification images of the correspondent boxed area. Scale bar, 100μm.

(j-m) Representative three-dimensional images of the different parts of the single small-size tumor, reconstructed from the pictures taken using a confocal microscopy. G; GFP, C; mCerulean, O; mOrange, Ch; mCherry.


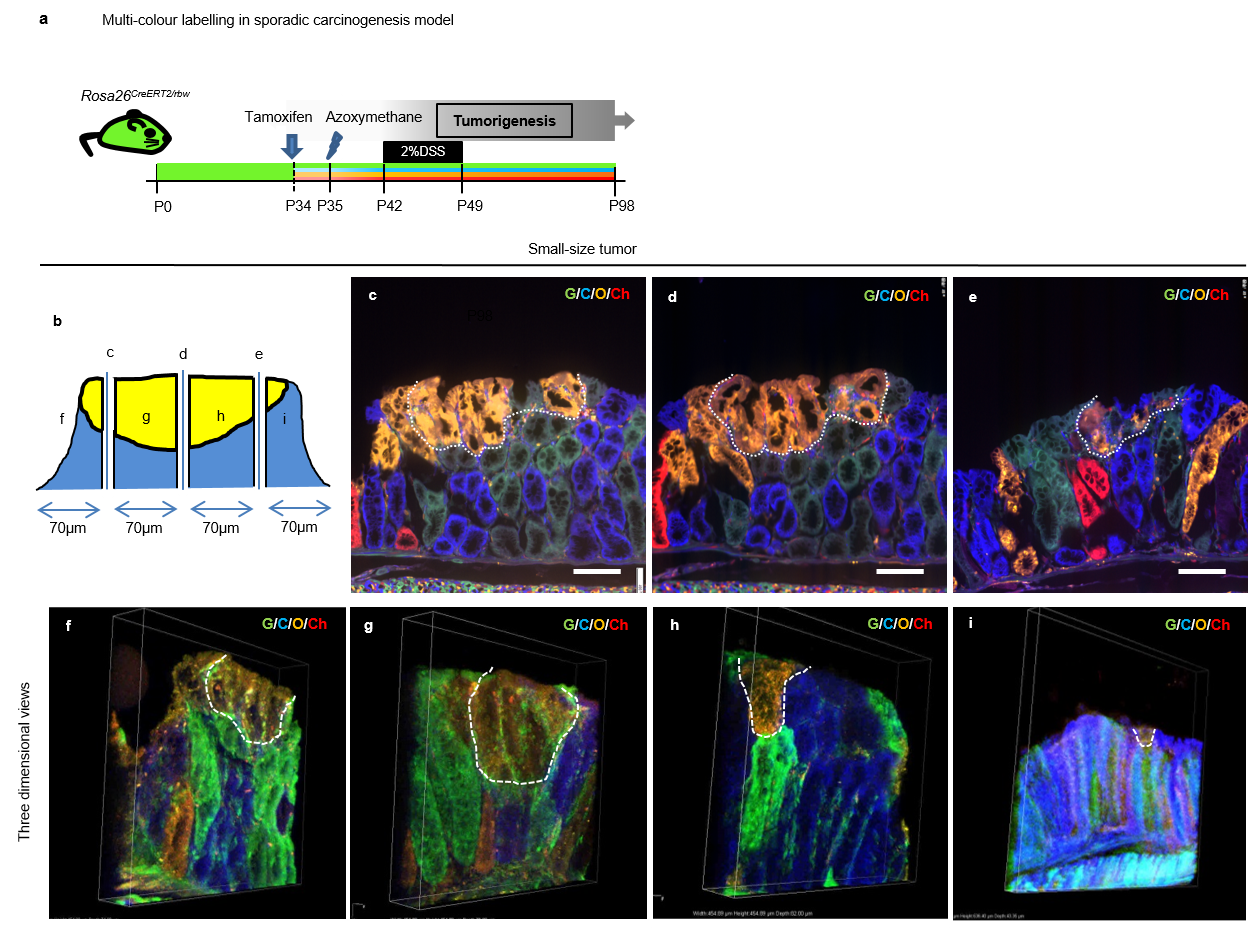


**Supplementary Figure 9 Monoclonal carcinoma that occurred in sporadic carcinogenesis mouse model was analyzed using multi-colour labeling method.**

(a) Schematic protocol of the CreERT2-mediatid ubiquitous multi-colour labeling in sporadic carcinogenesis model using *Rosa26 ^CreERT2/rbw^ ; APC^min/+^* mice. P, Postnatal day.

(b) Schematic representation of the slice preparation from small-size tumor for fluorescent image (c-e) and three-dimensional analysis (f-i) of traced cell-lineage. Yellow-colored area represents the tumor area consisting of the cells derived of a single mOrange-labeled cell; blue area represents surrounding normal tissues consisting of the cells derived of a single mCerulean-labeled cell.

(c-e) Representative fluorescent images the different parts of the single small-size tumor are shown. Scale bar, 100μm.

(f-i) Representative three-dimensional images of the different parts of the single small-size tumor, reconstructed from the pictures taken using a confocal microscopy. G; GFP, C; mCerulean, O; mOrange, Ch; mCherry.


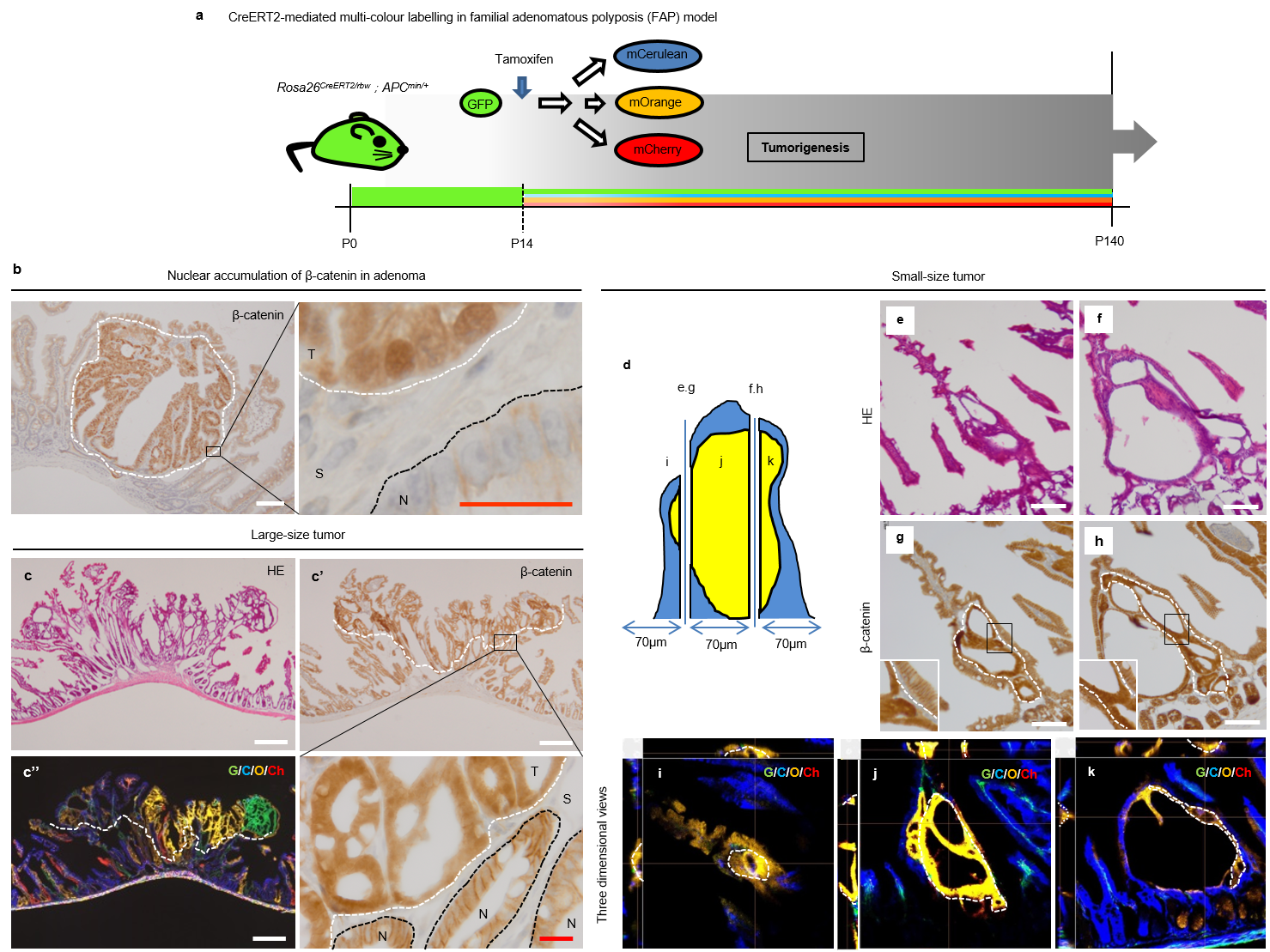


**Supplementary Figure 10 Clonal analysis thorough the process of familial adenomatous polyposis (FAP) formation using multi-colour labeling method.**

(a) Schematic protocol of the CreERT2-mediatid ubiquitous multi-colour labeling in familial adenomatous polyposis (FAP) model using *Rosa26^CreERT2/rbw^ ; APC^min/+^* mice. FAP spontaneously occurs in *APC^min/+^* mice before the age of 140. 140-day-old mice were analyzed. P, Postnatal day.

(b) The definition of tumor region. The area occupied by the cells in which nuclear accumulation of β-Catenin was detected was defined as tumor region. Representative β-Catenin (brown) staining of adenoma tissue (left). Scale bar, 100μm. Enlarged image of the boxed area is also shown (right). White dotted line and black dotted line represent the border between tumor and stromal area, and the border between normal and stromal area, respectively. T, tumor; S, Stroma; N, Normal. Scale bar, white; 100μm, red; 20μm.

(c) Reprsentative HE staining (c) and fluorescent image (c’’) of large-size tumor are shown. G, GFP; C, mCerulean; O, mOrange; Ch, mCherry. Representative β-Catenin (brown) staining (c’) and a high-power view of the boxed area (lower-right) are shown. White dotted line and black dotted line represent the border between tumor and stromal region and the border between normal epithelium and stroma region, respectively. T, tumor; S, Stromal; N, Normal. Scale bar, white; 100μm, red; 20μm.

(d) Schematic representation of the slice preparation from small-size tumor for HE staining (e, f), immunostaining of β-Catenin (g,h) and three-dimensional analysis of traced cell-lineage (i-k). Yellow-colored area represents the tumor area consisting of the cells derived of a single mOrange-labeled cell; blue area represents surrounding normal tissues consisting of the cells derived of a single mCerulean-labeled cell.

(e, f) Representative images of HE staining using two different slices prepared from the single small-size tumor at the interval of 70μm. Scale bar, 100μm.

(g, h) Representative images of β-Catenin (brown) staining using two different slices prepared from the single small-size tumor at the interval of 70μm. The area circled with white dotted line represents tumor area, evaluated by nuclear accumulation of β-Catenin. Insets represent higher magnification images of the correspondent boxed area. Scale bar, 100μm.

(i-k) Representative three-dimensional images of the different parts of the single small-size tumor, reconstructed from the pictures taken using confocal microscopy. G; GFP, C; mCerulean, O; mOrange, Ch; mCherry.


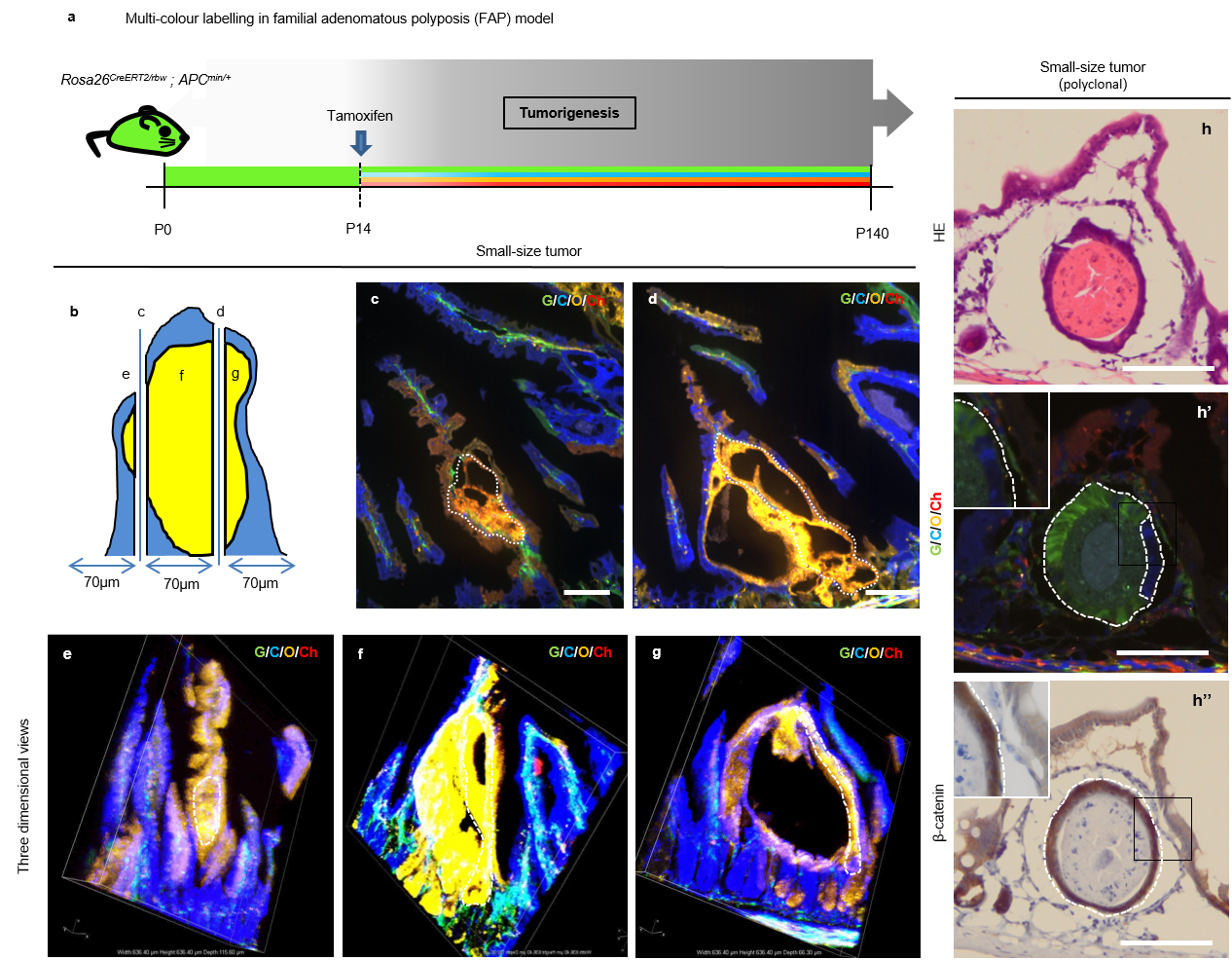


**Supplementary Figure 11 Lineage tracing using tamoxifen-inducible multi-colour labeling system to track the cell-fate thorough the process of familial adenomatous polyposis (FAP) formation.**

(a) Schematic protocol of the CreERT2-mediatid ubiquitous multi-colour labeling in familial adenomatous polyposis (FAP) model using *Rosa26 ^CreERT2/rbw^ ; APC^min/+^* mice. P, Postnatal day.

(b) Schematic representation of the slice preparation from small-size tumor for fluorescent image (c, d) and three-dimensional analysis (e-g) of traced cell-lineage. Yellow-colored area represents the tumor area consisting of the cells derived of a single mOrange-labeled cell; blue area represents surrounding normal tissues consisting of the cells derived of a single mCerulean-labeled cell.

(c, d) Representative fluorescent images of the different parts of the single small-size tumor are shown. Scale bar, 100μm.

(e-g) Representative three-dimensional images of the different parts of the single small-size tumor, reconstructed from the pictures taken using a confocal microscopy. G; GFP, C; mCerulean, O; mOrange, Ch; mCherry.

(h) Representative HE staining (h) and fluorescent image (h’) of small-size polyclonal tumor are shown. G; GFP, C; mCerulean, O; mOrange, Ch; mCherry. Representative β-Catenin (brown) staining (h’’) and a high-power view of the boxed area are shown as inset. White dotted line and black dotted line represent the border between tumor and stroma, and the border between normal epithelium and stroma, respectively. T, tumor; S, Stroma; N, Normal. Scale bar, 100μm.


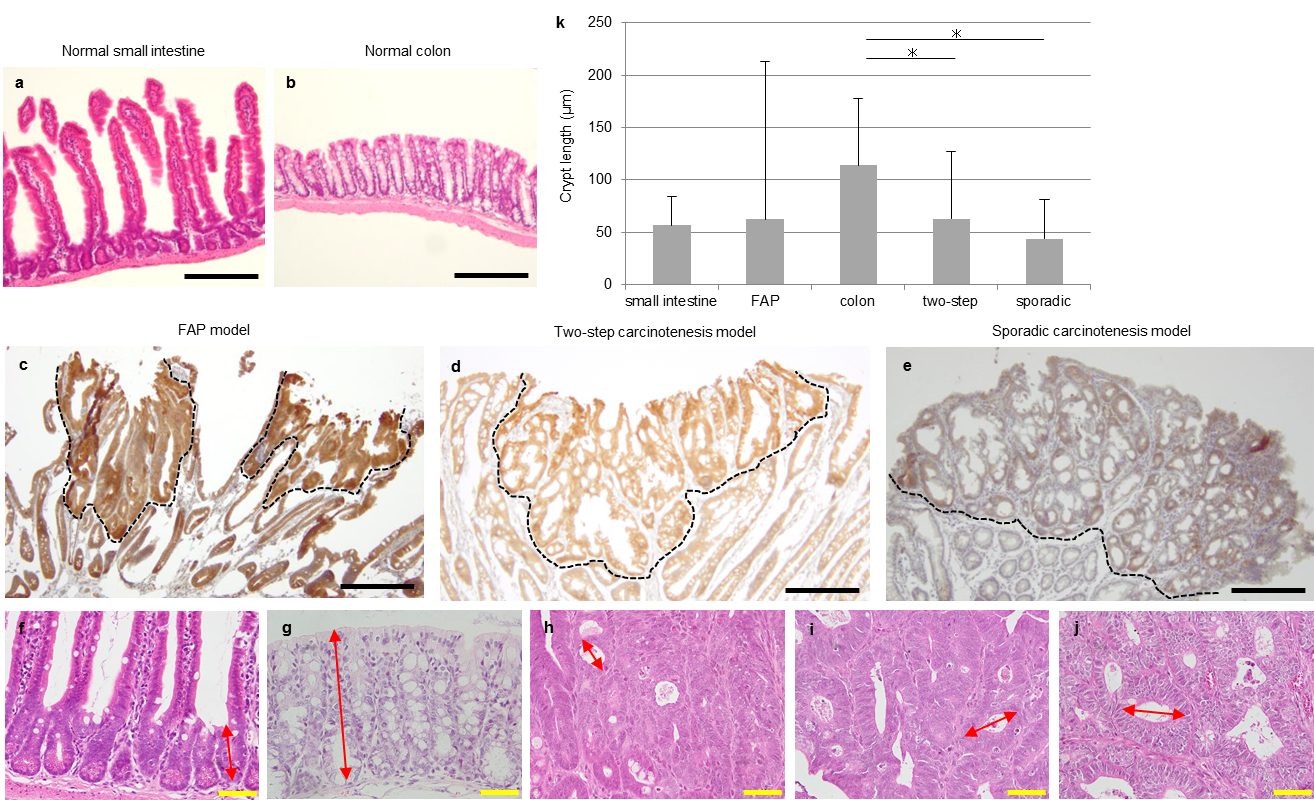


**Supplementary Figure 12 Comparison of crypt length in tumors with normal counterpart suggested that tumor development had been mediated by crypt fission.**

(a, b) Representative HE-staining of normal small intestine (a) and normal colon (b). Scale bar, 200μm.

(c-e) Representative β-Catenin (brown) staining of tumor in FAP model mice (c), two-step carcinogenesis model mice (d), and sporadic carcinogenesis model mice (e). The area circled with black dotted line represents tumor area, evaluated by nuclear accumulation of β-Catenin. Scale bar, 200μm.

(f-j) Representative images to explain how crypt length was measured in normal small intestine (f), normal colon (g), tumors that arose in FAP model mice (h), two step model mice (i), and sporadic model mice (j), respectively. Red double-headed arrows indicate the crypt length. Scale bar, 50μm.

(k) The crypt length of normal small intestine, small intestinal tumor (FAP), normal colon, and colon tumor (two-step carcinogenesis model and sporadic carcinogenesis model). *; p<0.05
